# Interface between breast cancer cells and the tumor microenvironment using platelet-rich plasma to promote tumor angiogenesis - influence of platelets and fibrin bundles on the behavior of breast tumor cells

**DOI:** 10.18632/oncotarget.15170

**Published:** 2017-02-09

**Authors:** Sheila Siqueira Andrade, Joana Tomomi Sumikawa, Eloísa Dognani Castro, Fabricio Pereira Batista, Edgar Paredes-Gamero, Lilian Carolina Oliveira, Izabel Monastério Guerra, Giovani Bravin Peres, Renan Pelluzzi Cavalheiro, Luiz Juliano, Afonso Pinto Nazário, Gil Facina, Siu Mui Tsai, Maria Luiza Vilela Oliva, Manoel João Batista Castello Girão

**Affiliations:** ^1^ Department of Gynecology of The Federal University of São Paulo, Brazil; ^2^ Department of Biochemistry of The Federal University of São Paulo, Brazil; ^3^ Department of Biophysics of The Federal University of São Paulo, Brazil; ^4^ Charitable Association of Blood Collection – COLSAN, São Paulo, SP, Brazil; ^5^ Cell and Molecular Biology Laboratory, Center for Nuclear Energy in Agriculture CENA, University of São Paulo USP, Piracicaba, SP, Brazil

**Keywords:** platelets, breast cancer, platelet-rich plasma, cancer, tumor microenvironment

## Abstract

Cancer progression is associated with an evolving tissue interface of direct epithelial-tumor microenvironment interactions. In biopsies of human breast tumors, extensive alterations in molecular pathways are correlated with cancer staging on both sides of the tumor-stroma interface. These interactions provide a pivotal paracrine signaling to induce malignant phenotype transition, the epithelial-mesenchymal transition (EMT). We explored how the direct contact between platelets-fibrin bundles primes metastasis using platelet-rich plasma (PRP) as a source of growth factors and mimics the provisional fibrin matrix between actively growing breast cancer cells and the tumor stroma. We have demonstrated PRP functions, modulating cell proliferation that is tumor-subtype and cancer cell-type-specific. Epithelial and stromal primary cells were prepared from breast cancer biopsies from 21 women with different cancer subtypes. Cells supplemented with PRP were immunoblotted with anti-phospho and total Src-Tyr-416, FAK-Try-925, E-cadherin, N-cadherin, TGF-β, Smad2, and Snail monoclonal antibodies. Breast tumor cells from luminal B and HER2 subtypes showed the most malignant profiles and the expression of thrombin and other classes of proteases at levels that were detectable through FRET peptide libraries. The angiogenesis process was investigated in the interface obtained between platelet-fibrin-breast tumor cells co-cultured with HUVEC cells. Luminal B and HER2 cells showed robust endothelial cell capillary-like tubes *ex vivo*. The studied interface contributes to the attachment of endothelial cells, provides a source of growth factors, and is a solid substrate. Thus, replacement of FBS supplementation with PRP supplementation represents an efficient and simple approach for mimicking the real multifactorial tumor microenvironment.

## INTRODUCTION

Promotion of tumor progression results from the establishment of an interface between cancer cells and their nearby stroma [1, 2]. In primary carcinomas, these heterotypic interactions are pivotal to induce an epithelial-mesenchymal transition (EMT), a process that alters the carcinoma's phenotype and plasticity and leads to malignant progression [3]. Several studies focus on this intriguing process in order to explain the effective trigger for efficient metastasis [1, 4–7]. However, it is extremely difficult to understand the exact cell-specific contribution of tumor–stromal interactions in the development of this structure–function relationship in cancer progression *in vivo* [2, 8]. In breast tumors, which are highly heterogeneous and result in a multifactorial disease [9–12], the cell-cell contact is key to triggering the metastasis process. Starting from this premise, we developed a platelet-rich plasma PRP-interaction-cell-based analysis in a cohort of breast tumors before and after PRP supplementation. We analyzed epithelial and stromal breast tumor cells extracted from 21 mammary biopsies from patients with different breast cancer subtypes in the presence of platelets and network of fibrin bundles to mimic the tumor-associated stroma. This included cells from biopsies of fibroadenoma and phyllodes fibroepithelial neoplasms, which are benign breast tumors [13]; cells from malignant breast tumors classified on the expression of estrogen (ER) and progesterone (PR) receptors; and HER2 classified into ER+, HER2+, subtypes luminal A and B, and HER2+ [14, 15]. We established heterotypic cell–cell contact and long/short-range diffusion of soluble factors using co-culturing methods that mimic the stroma as a supportive framework of the tumor condition containing fibrous proteins, e.g. fibrin(ogen), and growth factors from platelets. We also found that platelets and primary breast cancer cells collaborated in promoting the formation of capillary-like structures in endothelial cells that differs between subtypes of breast cancer. Although interactions between breast tumor cell lines have been described [2, 16–21], an understanding of how platelets and the network of fibrin bundles promote changes in the behavior of primary breast tumor cells in distinct subtypes of breast cancers is very limited. In this scenario, the main challenge was acquiring robust answers about host cell-to-host cell interactions that may determine the formation of pro-metastatic microenvironments. This behavioral heterogeneity affects treatment approaches and the development of experimental models that can provide relevant and reliable results in clinical trials.

## RESULTS

### Transfer of human mammary epithelial and stromal cells in monolayer cultures

Human breast epithelial cells and their respective stromal cells from benign and malignant breast tumors, derived from mastectomy (partial or total) specimens and freshly isolated as terminal ductal organoids, were grown exponentially for 10 to 12 days and generated confluent monolayers on the plastic surface in primary cultures. The initial stage of cell growth was termed passage 1 (p1). To expand or freeze (in vapor phase in liquid Nitrogen), epithelial and stromal cells were harvested by trypsin and EDTA release. When cryopreserved, single cells were reactivated, 85% were viable, and grew out successfully in culture at appropriate cell densities (data not shown).

**Figure 1:**
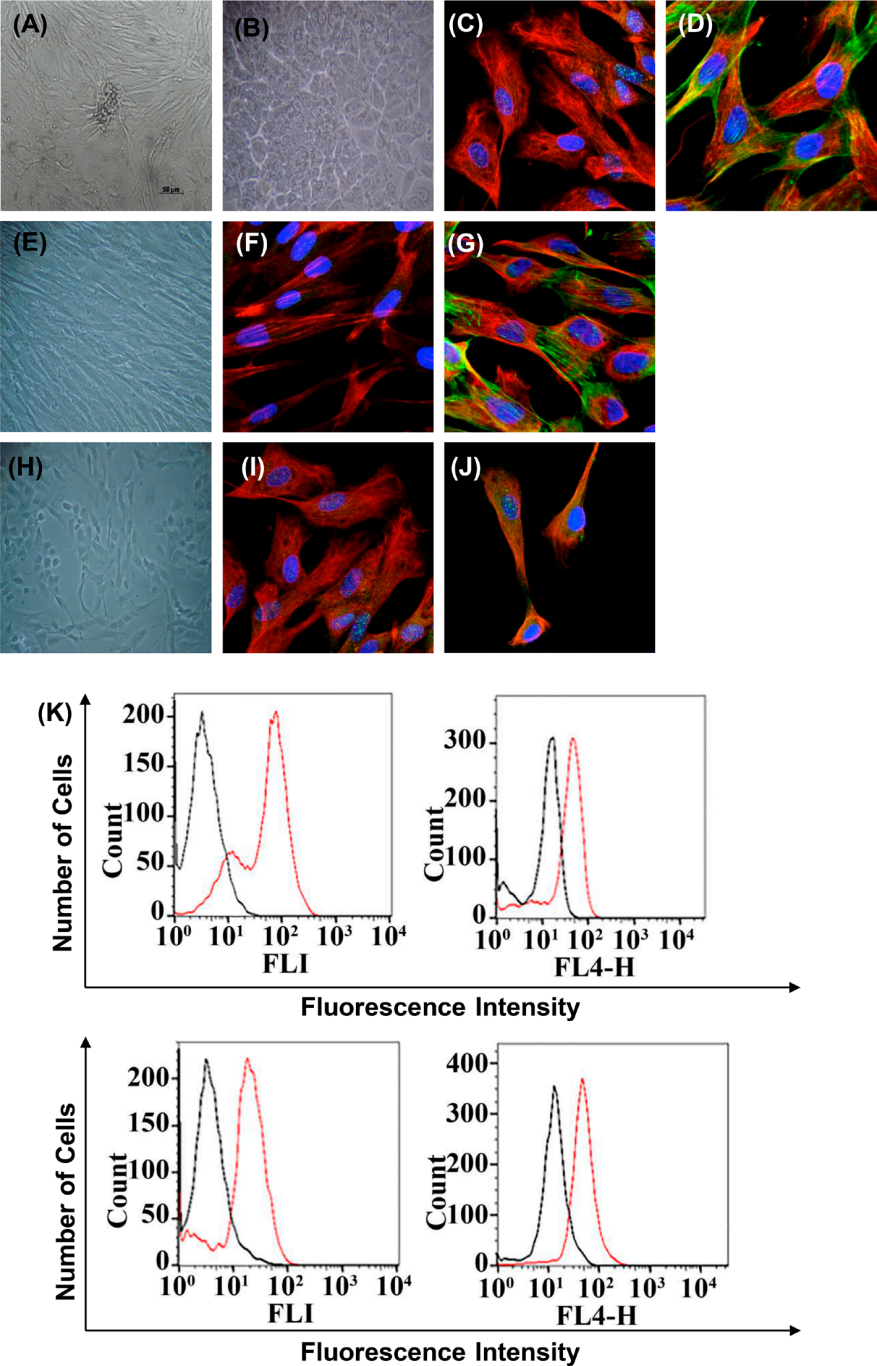
Immunophenotype of breast cancer cells from patients with fibroadenoma and phyllodes tumors (non-cancerous breast condition), luminal A and B, and HER2+ breast cancer subtypes used in all experiments Cells are shown under phase contrast microscopy and indirect immunofluorescence for vimentin, cytokeratin, E-cadherin, N-cadherin, PAI-1, claudin 1, phalloidin, and DAPI (blue for nuclei). (**A**) Phase contrast–confluent culture of organoid tumor cells after 3 days. (**B**) Confluent epithelial breast tumor cells after 2 days in culture. Mycoplasma contamination was not observed in any of the processed tissues. (**C**–**D**) Analysis of epithelial and mesenchymal markers by confocal microscopy; cytokeratin, vimentin, and phalloidin. (**E**) Confluent stromal breast tumor cells after 2 days in culture. (**F**–**G**) Positivity for the mesenchymal marker vimentin by confocal microscopy. We observed the cytoskeletal organization pattern when using phalloidin. (**I**–**J**) Breast cancer cells in epithelial-mesenchymal transition. Analysis of epithelial and mesenchymal markers by confocal microscopy; cytokeratin, vimentin, and phalloidin-cytoskeleton. (**K**) Flow cytometry histograms representative of E-cadherin, N-cadherin, PAI-1, and claudin 1 on tumor cells with EMT. The histogram on the left represents a control staining using an isotype-matched control antibody. These experiments were performed with cultured cells from all specimens collected from patients.

The morphological characteristics of epithelial and stromal cells (fibroblast) were evaluated; epithelial cells showed flattened and polygonal shape, and stromal cells showed a fibroblastic shape with large size and long cell protrusions in both poles. With increasing confluence, epithelial cells exhibited a more prominent polygonal shape, and stromal cells exhibited a spindle-like shape; both cell types grew in homogeneous cell populations (Figure 1A, 1B, 1E, and 1H). The characterization of cells was conducted by immunolocalization by confocal microscopy and fluorescence-activated cell sorting. The cells obtained in the first step of differential centrifugation presented the epithelial phenotype with positive cytokeratin-18 and negative vimentin (Figure 1B–1D). The stromal cells obtained in the last step of differential centrifugation showed the fibroblastic phenotype with positive vimentin (Figure 1E–1G). Finally, the epithelial and mesenchymal markers involved in EMT were also detected in some of the epithelial cells from patients with luminal B and HER2+ subtypes of breast carcinoma. The analysis indicated that cytokeratin-18 (epithelial marker) and vimentin (fibroblast marker) were colocalized on the cell surface (Figure 1H–1J). In addition, the E-cadherin levels were also reduced in comparison with N-cadherin when analyzed by fluorescence-activated cell sorting (Figure 1K). The plasminogen activator inhibitor-1 (PAI-1; Serpine 1, mesenchymal marker) was consistently detected as upregulated; the claudin 1 epithelial marker was also consistently detected as downregulated (Figure 1K). The characteristics of 21 tumor specimens collected from patients with different subtypes of breast carcinoma were previously confirmed (Table 1). Mycoplasma contamination was not observed in any of the processed tissues.

**Table 1:** Characteristics of samples used in the study

Groups	Samples	Menopausal status	Hormonal status (ERα−ERβ)	Hormonal status (PR)	HER2	Ki-67 expression (percent) < 10% ≥ 10%	Histological grade
**Fibroadenoma**	1 2 3	Premenopausal Premenopausal Premenopausal	N/A N/A N/A	N/A N/A N/A	N/A N/A N/A	< 10% < 10% < 10%	unspecific unspecific unspecific
**Phyllodes**	4 5 6	Premenopausal Premenopausal Menopausal	+ ERβ + ERβ −	− − −	− − −	≥ 17.2% ≥ 27.4% < 10%	N/A N/A N/A
**Luminal A**	7 8 9 10	Menopausal Postmenopausal Postmenopausal Postmenopausal	+ ERα + ERα + ERα + ERα	+ + + +	− − − −	≥ 25.4% ≥ 19.7% ≥ 21.5% ≥ 27.1%	Grade 2 Grade 1 Grade 1 Grade 3
**Luminal B**	11 12 13 14 15 16 17	Menopausal Menopausal Postmenopausal Premenopausal Premenopausal Premenopausal Premenopausal	+ ERα + ERα + ERα + ERα + ERα + ERα + ERα	+ + + + + + +	+ + − + − − −	≥ 43.3% ≥ 66.7% ≥ 33.1% ≥ 57.9% ≥ 50.8% ≥ 31.2% ≥ 32.8%	Grade 2 Grade 3 Grade 1 Grade 3 Grade 2 Grade 1 Grade 2
**HER2**	18 19 20 21	Menopausal Premenopausal Postmenopausal Postmenopausal	− − − −	− − − −	+ + + +	≥ 61.8% ≥ 71.2% ≥ 56.0% ≥ 66.7%	Grade 3 Grade 3 Grade 2 Grade 3

N/A = Not available. *Age range 34–67 years.

### Mimicking the network of fibrin bundles in tumor cells with the replacement of FBS by platelet-rich plasma

We first examined the abilities of epithelial and stromal tumor cells to support platelets and the formation of network fibrin bundles in contact with normal pooled PRP. We analyzed epithelial and stromal cells from tumor biopsies of 21 patients with and without breast cancer including 3 fibroadenomas, 3 Phyllodes, 4 luminal A, 7 luminal B, and 4 HER2+ tumors representing the major breast tumor subtypes. TNBC (triple-negative breast cancer) was not included in this study because it was not found in the sample set [14] (Table 1).

**Figure 2:**
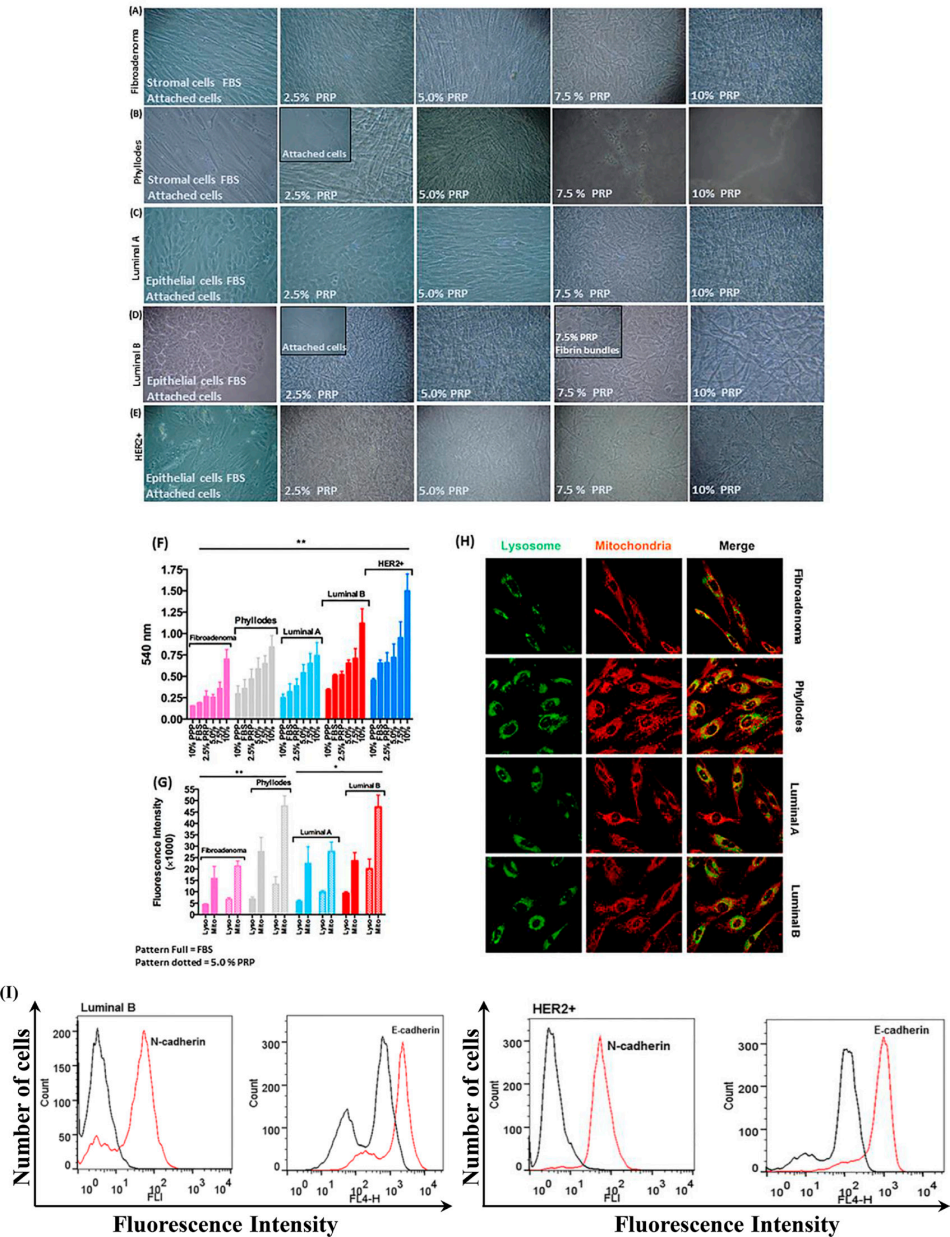
Morphology of stromal and epithelial cells during gradual PRP supplementation (**A**–**B**) The primary culture of stromal breast tumor cells from patients with fibroadenoma and phyllodes benign breast tumors developed more spindle-shaped morphology and proliferated faster than cells cultured in FBS. (**C**–**E**) Primary culture of epithelial breast tumor cells from patients with luminal A and B and HER2+ breast cancer subtypes developed more prominent polygonal shape, proliferated faster, and migrated in higher amounts from the plastic surface to fibrin bundles than cells cultured in FBS. (**F**) Cell viability by the MTT assay. Stromal and epithelial breast tumor cells (≈2 × 10^3^ densities) were cultured in the appropriate medium without phenol red and supplemented with specifically 2.5% PRP for 24 h in 96-well microtiter plates. (**G** and **H**) MitoTracker Orange, LysoTracker Green, and DAPI fluorescent micrographs of stromal and epithelial breast tumor cells specifically supplemented with 5.0% PRP. Total lysosomal and mitochondrial amount. Scale bar = 20 μm. Quantification of fluorescence intensity of LysoTracker and MitoTracker in living cells to evaluate the number of lysosomes and mitochondria numerically in each cell. (**I**) Representative flow cytometry histograms of E-cadherin and N-cadherin on HER2+ epithelial breast tumor cells with EMT supplemented with PRP. The histogram on the left represents a control staining using an isotype-matched control antibody. These experiments were performed with cultured cells from all specimens collected from patients.

Morphological characteristics and the formation of network fibrin bundles were assessed based on phase-contrast microscopy. As we gradually introduced PRP, changes in cell morphology in both epithelial and stromal breast tumor cells were observed. In addition, the cell culture medium converted from liquid to a gel-like consistency (rapidly < 30 minutes) as the result of the formation of the network of fibrin bundles. The initial gel formation was tumor-subtype and cancer cell-type-specific, and the concentration of PRP was decisive in this process (Figure 2A–2E and Supplementary Figure 1); moreover, the addition of PRP at the highest concentration studied (10%) to a cell-free system did not show the formation of any gel-like material. The replacement of FBS with PRP clearly demonstrated that the cells proliferated at a substantially faster rate, mainly at the highest concentrations of platelets and fibrin bundles (5.0 to 10% PRP). Both epithelial and stromal breast tumor cells migrated from the plastic surface to the new and solid substrate of fibrin bundles after the introduction of PRP in the culture medium (Figure 2A–2E). However, stromal cells extracted from patients with fibroadenoma and patients with phyllodes benign breast tumors presented very different proliferation rates. Those from fibroadenoma biopsies developed the network of fibrin bundles in the presence of 7.5% PRP; conversely, stromal cells from phyllodes tumors showed a significantly faster onset and rate of formation of the network of fibrin bundles in the presence of 2.5% PRP (Figure 2A and 2B). In addition, significant differences in the proportion of Ki67+ cells in both conditions were observed before the introduction of PRP in which phyllodes tumor cells showed the frequency of cells positive for Ki67 higher than fibroadenoma stromal cells (Table 1). Furthermore, the proliferation rate of stromal cells from patients with phyllodes tumors was approximately twofold higher than that of stromal cells from women with fibroadenoma in the 7.5% PRP supplementation as evaluated by the MTT assay (Figure 2F). Phyllodes stromal cells were increasingly organized as clumped cells in the fibrin network with increasing PRP concentrations (Figure 2B). The platelet-poor plasma (PPP) was used as a control and revealed a decreased metabolic activity in the absence of platelet-treated cells (data not shown). The presence of PRP might have induced perturbations in cell metabolism because of anoikis triggered by the loss or changes in the anchoring and autophagic impairment that induce EMT [22]. We, therefore, investigated the possible increase in the amount of lysosomes that leads to the accumulation of aberrant mitochondria and its association with phenotype transition [23]. The amounts of lysosome and mitochondria were increased in stromal cells from phyllodes tumor as observed using LysoTracker and mitoTracker staining (Figure 2G and 2H). We determined the intensity fluorescence of LysoTracker and MitoTracker in living cells to numerically evaluate the amounts of lysosomes and mitochondria in each cell. These amounts were about twofold greater in phyllodes stromal cells than stromal cells from fibroadenoma biopsies, both cultured in 5.0% PRP (Figure 2G). Supplementation with 5% PRP was used for confocal microscope observation because cells were spread and attached in both cases.

The epithelial cells from luminal (A and B) and HER2+ breast tumors responded differently to PRP supplementation. Luminal A breast cancer subtype cells showed high proliferation in the gradual contact with PRP compared with gradual FBS supplementation (Figure 2C). Epithelial cells from patients with luminal B breast cancer subtype changed anchoring or adhesion from the plastic surface to the early fibrin bundles in 2.5% PRP (Figure 2D). This may be related to the high frequency of cells positive for the Ki67 proliferation marker and the EMT process detected in some of the luminal B cells analyzed (*n* = 3, total *n* = 7 luminal B) before the introduction of PRP (Table 1), which was exacerbated by the presence of platelets and fibrin bundles. We observed an increase in the metabolic activity of luminal A and B epithelial cells at 24 h in the same conditions. However, luminal B cells increased their metabolic activity by a factor of ≈ two higher than luminal A epithelial cells in 10% PRP (Figure 2F). In agreement with these results, the amounts of lysosome and mitochondria in epithelial cells from luminal B tumors were twice as high as those in epithelial cells from luminal A (Figure 2G and 2H). When we discarded the luminal B epithelial cells in the EMT process (Figure 1H–1J) and only analyzed cells with an epithelial phenotype, we observed cell migration and adhesion to fibrin bundles, which may have triggered the EMT process in the presence of platelets (Figure 2I) [4].

Epithelial cells from patients with HER2+ breast tumors showed higher metabolic activity in all PRP concentrations compared with epithelial cells from luminal A and B breast tumors (Figure 2F). Our results showed that the fraction of Ki67+ cells was higher in all cell-HER2+ tumors (Table 1). However, the amounts of lysosome and mitochondria were not evaluated in epithelial HER2+ cells because these cells were not attached in 5.0% PRP and minimal cells were present on the plastic surface in 2.5% PRP. These results suggest that the increase in metabolic and motility capacity of HER2+ cells might result from the influence of platelets that induced conversion from the epithelial to mesenchymal cellular phenotype with a change in the expression of their respective markers, E-cadherin and N-cadherin, as shown in Figure 1K and 2I.

### Cancer cell-type-specific and tumor-subtype differences in proteolytic activity

We analyzed pre- and post-tumor cells in culture with PRP supplementation to investigate the relationships between the intra-tumor-cell proteolytic activity and PRP supplementation. The screening and partial characterization of the hydrolytic activity of proteases in the CM of primary breast tumor cells were conducted using the thrombin substrate, combinatorial libraries of internally quenched fluorescence substrates, and gel zymography.

It is known that tumor and inflammatory cells secrete proteolytic enzymes, proteases such as serine (thrombin-like and trypsin-like), metallo- (Matrix metalloproteases), and cysteine proteases (cathepsins-cysteine proteases) that can activate platelets by inducing alterations in the paracrine signaling manner and formation of the fibrin network with consequent support for thrombin generation [24–27]. In addition, platelets are a source of thrombin and PRP (platelet-rich plasma) contain low concentrations of fibrinogen and fibrin, plasma enzymes, e.g., coagulating factors that are mostly serine proteases, which interfere with spontaneous platelet aggregation and the formation of fibrin network [27–29].

**Figure 3:**
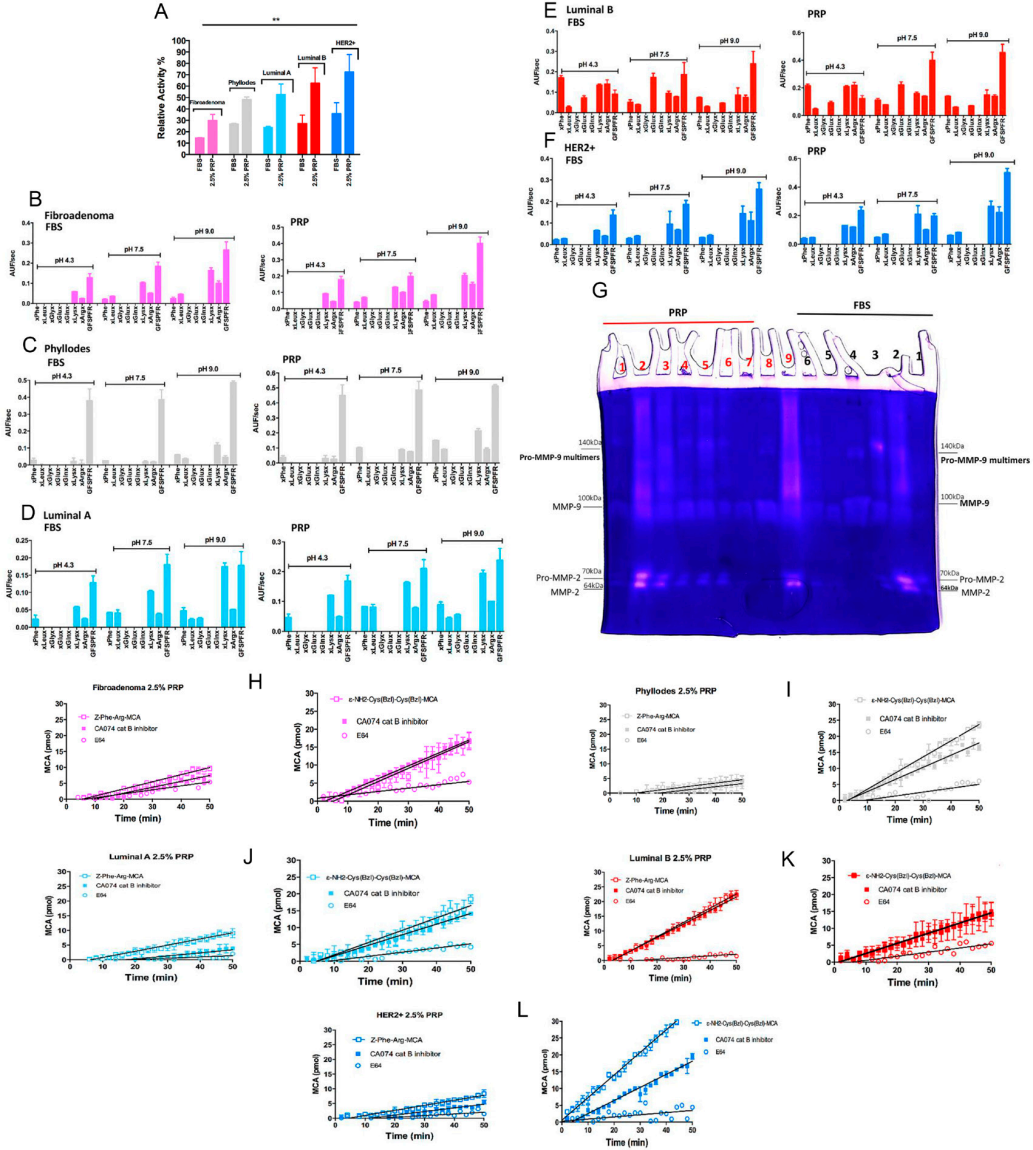
Detection of proteolytic activity on CM with 2.5% PRP supplementation of stromal and epithelial breast tumor cells (**A**) Evaluation of degradation of the Benzoyl-Phe-Val-Arg-MCA substrate, a specific substrate for α-thrombin. Epithelial and stromal-CM with 2.5% PRP supplementation were compared with FBS supplementation (both conditions were incubated at 37°C for 16 h). (**B**–**F**) Screening of endopeptidase activities presents in epithelial and stromal-CM with PRP supplementation. The rate of hydrolysis of the Abz-GXXZXXQ-EDDnp peptide sublibraries by epithelial and stromal-CM is shown under three pH conditions: 4.3, 7.5, and 9.0. The endopeptidase activity was measured for each sublibrary in which “Z” was either phenylalanine (XPheX), leucine (XLeuX), glycine (XGlyX), glutamate (XGluX), glutamine (XGlnX), lysine (XLysX), or arginine (XArgX). The velocity of hydrolysis was measured as Arbitrary Units of Fluorescence (AUF)/sec. (**G**) Zymography for MMP-2 and MMP-9 in epithelial and stromal-CM cells isolated from women with breast cancer. Cell culture in monolayers and co-cultures (stromal and epithelial breast tumor cells from the same patient). Samples: FBS (black) – 1) Fibroadenoma, 2) Phyllodes, 3) Luminal A, 4) Luminal B co-culture, 5) Luminal B, 6) HER2+; PRF (red) 1) Fibroadenoma, 2) Phyllodes, 3) Luminal A (2 × concentrated – 20 μg), 4) Luminal A (1 × concentrated – 10 μg), 5) Luminal A co-culture, 6) Luminal B co-culture, 7) Luminal B, 8) HER2+ and 9) Her2+ co-culture. MMP-9 multimers formation was previously described [32]. (**H**–**L**) Cysteine protease activities in stromal and epithelial-CM cells from women with different breast cancer subtypes. Approximately 2 × 10^5^ cells were cultivated for 3 days; the enzymatic activity was measured in CM submitted to PRP supplementation (in the absence of phenol red) and removed from each well using stromal and epithelial cells; Z-FR-MCA (20 μM) (200 μL final volume) and stromal and epithelial cells; ε-NH2-caproyl-Cys(Bzl)- Cys(Bzl)-MCA (20 μM) in 200 μL final volume. A maximum of 10% substrate consumption was considered; each point represents the mean ± 95% confidence interval of two replicates. The presence of PMSF did not affect the total activity of proteases. Inhibitor concentrations: PMSF = 1 mM; CA074 = 1 μM; E64 = 5 μM. These experiments were performed with cultured cells from all specimens collected from patients.

Therefore, the proteolytic activity of thrombin in the CM of cultured breast tumor cells was determined by an enzymatic reaction in the presence of Benzoyl-Phe-Val-Arg-MCA, a specific substrate for α-thrombin. As depicted in Figure 3A, 2.5% PRP supplemented epithelial and stromal-CM incubated at 37°C for 16 h, showed twofold higher hydrolysis than with 10% FBS supplementation in all breast tumor cells that were tested with thrombin Benzoyl-Phe-Val-Arg-MCA substrate. These results demonstrate the effectiveness of the CM containing PRP supplementation to cleave the thrombin substrate; in addition, they demonstrate that epithelial and stromal breast tumor cells express detectable thrombin levels even with FBS supplementation. The PPP (platelet-poor plasma) supplementation was used as a control and indicated a lower ability to detect thrombin activity in both epithelial and stromal breast tumor culture mediums. This reinforces the observation that growth factors and platelets are involved in stimulating breast tumor cells to release and supply thrombin in the CM.

**Figure 5:**
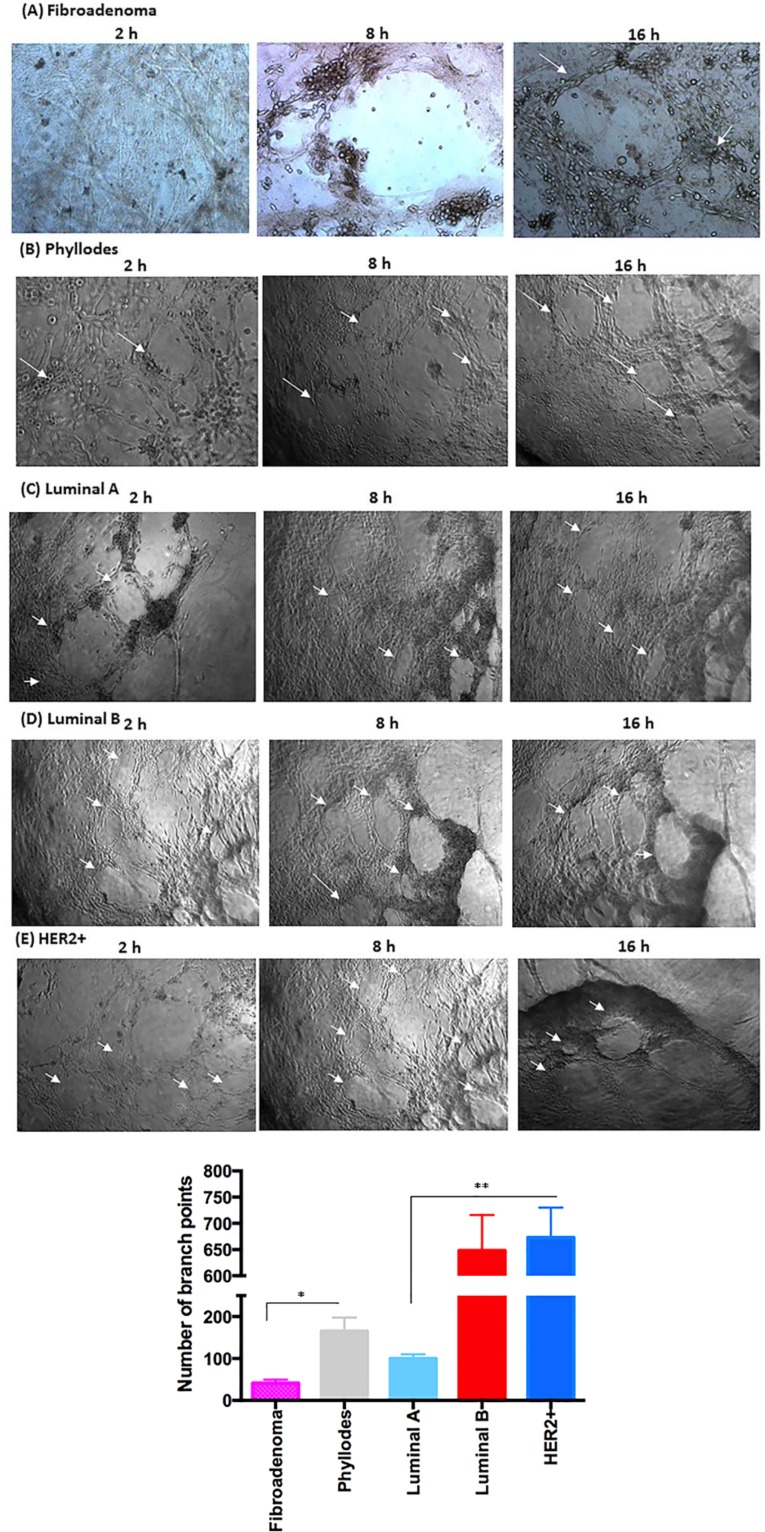
Co-culture of breast tumor cells under PRP supplementation and the formation capillary-structure tubes induced by HUVEC (**A**) Fibroadenoma stimulates minimally cluttered capillary tube formation. (**B**) Phyllodes showed enhanced angiogenesis-promoting ability. (**C**) Luminal A promoted a slight formation of angiogenic structures in HUVEC. (**D** and **E**) In luminal B and HER2+, the capillary tube structure was stable and defined, and 6.5-fold higher than luminal A. Arrows indicate the formation of angiogenic structures. Quantification of HUVEC branch points over an 8–16 hour time course (see Methods). (**F**) Measurement of the angiogenic structure formation (per well). These experiments were performed with cultured primary cells from all specimens collected from patients. Significant differences versus controls are presented (ANOVA; *p ≤ 0.05, **p ≤ 0.001). Inverted microscope images (× 40 magnification). Scale bars 20 μm.

Moreover, using synthetic combinatorial libraries as substrates, we demonstrated that epithelial and stromal breast tumor cells have a higher capacity to secrete other proteolytic enzymes in the presence of 2.5% PRP than in the presence of 10% FBS. The Abz-G-XXZXX-QEDDnp sublibraries containing Phe, Leu, Gly, Glu, Gln, Lys, and Arg at fixed Z positions (Z = S1 subsite of endoproteinases [30]) were assayed when incubated with the CM at pH 4.3, 7.5, and 9.0. However, each condition and subtype of breast cancer present specific activities. The sublibraries containing Phe and Lys as the fixed residue were cleaved at similar levels at pH 7.5 and 9.0 in all analyzed CMs; hydrolysis was ≈ twice as high in the presence of PRP than FBS supplementation in all breast tumor cells extracted from patients (Figure 3B–3F). The protease activity with the highest hydrolytic rate at pH 9.0 in sublibraries containing Phe, Lys, and Arg was observed in the CM of phyllodes stromal cells, and luminal A and B epithelial cells; metalloproteinase and serine proteinase activities (not thrombin-like, [31] were detected at this pH using the Abz-GFSPFR-EDDnp bradykinin-derived substrate, which effectively cleaved the GFSPFR peptide at the Phe-Arg bond as confirmed by mass spectrometry (MS) (data not shown). The preference for Phe (at P1 position) substrates and the observed sensitivity to the metalloproteinase inhibitors ο-phe (ortho-phenanthroline), EDTA, and TLCK (trypsin-like serine proteinase inhibitor), but not the serine proteinase inhibitor TPCK (chymotrypsin-like serine proteinase inhibitor), can be biochemically interpreted as evidence of metalloproteinase and serine proteinase activity. This result was confirmed in the gel zymography that revealed gelatinolytic activities (Figure 3G); the breast tumor cells from fibroadenoma, phyllodes, luminal A, and HER2+ showed increased secretion of matrix metalloproteinase-2 and -9 (MMP-2 and MMP-9) following exposure to platelets, i.e., cultured with PRP supplementation. MMP-2 was poorly detected in cultures with FBS supplementation. Zymography in the CM from the co-cultured epithelial cell with respective stromal cells from each patient sample was performed to mimic the microenvironment: the zymograph in this condition revealed high concentrations of both MMPs when stromal cells were introduced to the culture system (Figure 3G).

However, proteinase activity in the CM of luminal B epithelial cells was observed in the sublibraries containing Phe, Glu, Lys, and Arg at pH 4.3, 7.5, and 9.0 with differences in intensity, and was 1.3-fold higher in the presence of 2.5% PRP supplementation compared to the other breast tumor cells (Figure 3E). The proteinase activity of the sublibrary containing Phe as the fixed residue showed the highest hydrolytic rate at pH 4.3 when using the CM with PRP supplementation. The same metalloproteinase and serine proteinase activities identified in other CMs of breast tumor cells in the presence of PRP were detected using Abz-GFSPFR-EDDnp as the substrate. However, the effect of class-specific peptidase inhibitors on the hydrolysis of Abz-GXX-Phe-XXQ-EDDnp by the conditioned medium from luminal B cells shows partial inhibition in the presence of ο-phe and EDTA only at pH 9.0. At pH 7.3, the activity was partially affected by TLCK and TPCK, the classical inhibitors of trypsin-like serine proteinase, indicating the presence of other proteases in the secretome of luminal B epithelial cells. Moreover, the zymographic analysis revealed the highest gelatinolytic activity of MMP-2 and -9 in the presence of PRP supplementation. Gelatinolytic activity was even more prominent in the co-culture system with luminal B stromal cells (Figure 3G).

Cysteine proteinase activity using Z-Phe-Arg-MCA (carbobenzoxy-Phe-Arg-7-amide-4-methylcoumarin) and ε-NH_2_-Cys(Bzl)-Cys(Bzl)-MCA was analyzed using papain-like cysteine proteinase and cat B substrates, respectively, with or without inhibitors (E64-5 μM and CA074 - 1 μM). Figure 3I and 3L show that cat B was a cysteine protease present in the CM with PRP supplementation of the breast tumor cells phyllodes and HER2+, exhibiting high hydrolytic activity on the ε-NH_2_-Cys(Bzl)-Cys(Bzl)-MCA substrate. Cat B was either not detected or only slightly detected using both substrates in fibroadenoma and luminal A and B cells, which implies another cysteine proteinase activity (Figure 3H, 3J, and 3K).

As expected, cysteine proteinase activity was detected in the CM with 2.5% PRP in luminal B epithelial cells, as demonstrated in our previous study [27]. This result supports the participation of other papain-like cysteine proteinase activity, confirmed by the E64 inhibition (Figure 3K).

Overall, the partial screening of hydrolytic activity confirms the presence of thrombin (with or without PRP supplementation), trypsin-like serine proteinases, MMP-2 and MMP-9 metalloproteinases, and cathepsin-cysteine proteinases in the secretome of breast tumor cells. Furthermore, it reveals increased and more evident proteolytic activities with PRP supplementation than with FBS supplementation, which might represent an approximation of the multifactorial tumor microenvironment.

### PRP induced alterations in the focal adhesion complex and changes in cell phenotype

**Figure 4:**
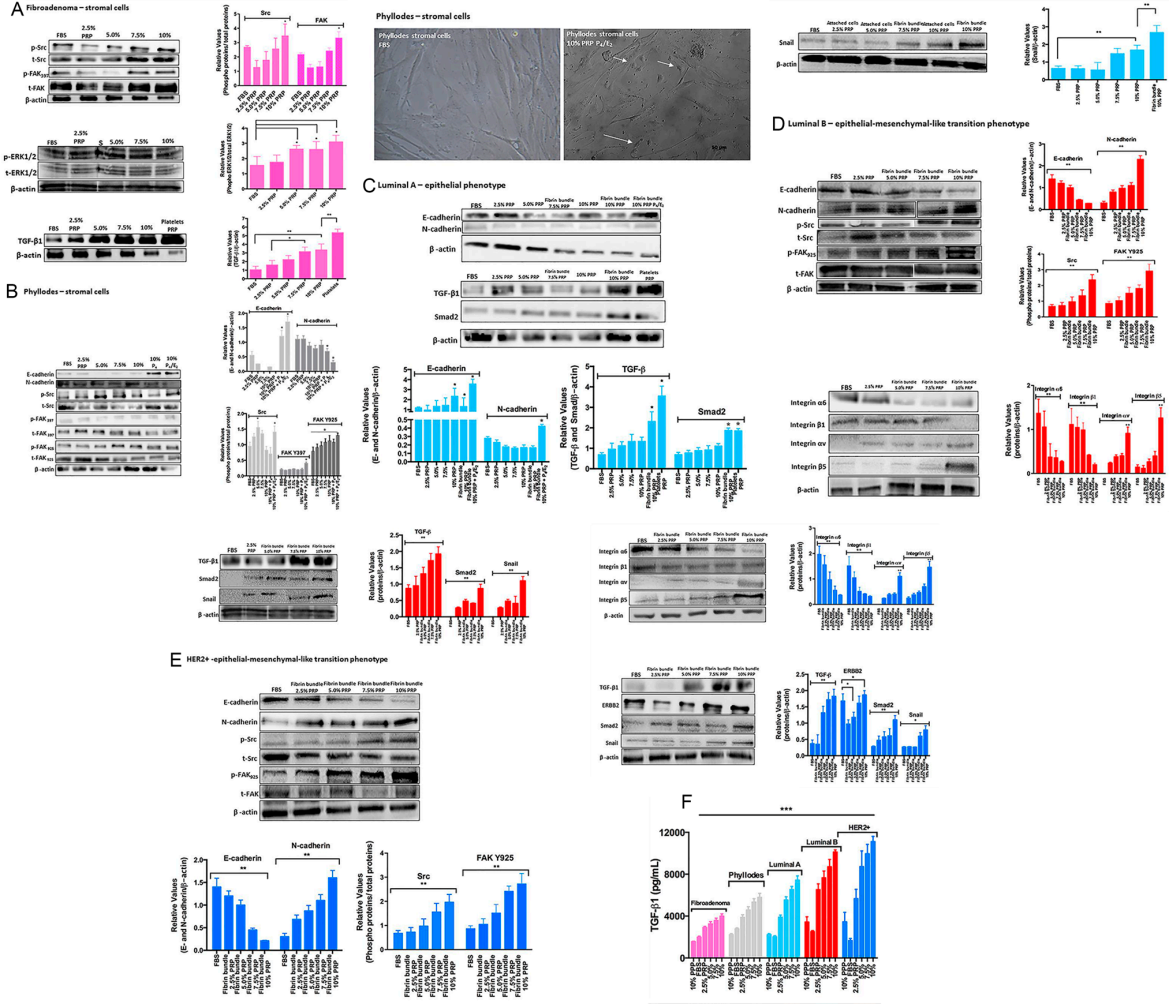
Stromal and epithelial breast tumor cells under PRP supplementation (including cells attached to the plastic surface and cells attached to the network of fibrin bundles) were assessed by immunoblotting (**A**) Fibroadenoma stromal cells under PRP supplementation showed increased phospho-Src, phosphos-FAK(Y)397, and phospho-ERK1/2, and upregulated TGF-β1 expression. The graph represents the densitometry analyses from immunoblotting results. (**B**) Phyllodes stromal cells under PRP supplementation showed EMT reversion and upregulation of E-cadherin and downregulation of N-cadherin expression after treatment with P4 and the E2+P4 combination. Phospho-Src and phospho-FAK(Y)925, but not FAK(Y)397, showed increased phosphorylation. Phase contrast–confluent culture of phyllodes stromal cells showed morphology alteration in the presence of steroid hormones under PRP supplementation. (**C**–**E**) Luminal A and B and HER2+ epithelial cells under PRP supplementation showed EMT process with downregulation of E-cadherin and upregulation of N-cadherin expression. Phospho-Src and phospho-FAK(Y)925, but not FAK(Y)397, showed increased phosphorylation, and the α6β1 integrin subunits showed decreased expression. Conversely, the αvβ5 integrin subunits showed increased expression. The analysis of the TGF-β, Smad2, and Snail metastasis markers showed increased expression in the highest PRP concentrations. The results are represented as band intensities in arbitrary units relative to the respective total load of total proteins and control (β-actin). The statistical significance was evaluated using one-way ANOVA followed by the Tukey's test. These experiments were performed with cultured primary cells from specimens collected from patients, *p ≤ 0.05, **p ≤ 0.001. (**F**) Concentration of total TGF-β in CM from breast tumor cells. The conditioned medium was collected, centrifuged to remove platelets, and the presence of TGFβ1 in the supernatant measured by ELISA. Each bar represents the mean ± SEM of *n* = 2–6. ***p < 0.001 as determined by Student's *t* test.

Like many other cell types, serum-adapted breast tumor cells show profound changes in their ability to grow and proliferate. Moreover, PRP supplementation mimics one of the critical structures in the tumor microenvironment, which is the interface between platelets and the network of fibrin bundles localized between actively growing breast cancer cells and tumor stroma [18]. The fibrin bundles form an extracellular matrix-like structure to which cells migrate. Our data showed that breast tumor cells supplemented with PRP induced the formation of a solid substrate containing platelets and fibrin bundles on the top of cell culture, which allowed migration of specific cells from the plastic surface to the fibrin bundles. Therefore, we investigated whether these selected breast tumor cells that adhered to the network fibrin bundles were guided by platelets and had their malignancy proprieties altered compared to the cells that remained adhered to the plastic surface. Thus, the FAK-Src focal adhesion complex pathway, the most important complex to convey survival signals in breast cancer cells [33], was initially investigated. Platelets-induced TGF-β subsequently revealed phenotype changes (EMT) in breast tumor cells, which caused phosphorylation of cytoplasmic Smad proteins and their dispatch to the nucleus, activating the transcription factor Snail [4, 34]. To investigate this issue, the detection of critical proteins by immunoblotting of lysates of epithelial and stromal cells from distinct breast tumor subtypes submitted to PRP supplementation was explored. We compared stromal cells from fibroepithelial neoplasms, fibroadenoma, and phyllodes breast tumors under PRP supplementation and observed that the phosphorylation of Src-Tyr-416 and FAK-Tyr-397 gradually increased with increasing concentrations of PRP supplementation in stromal cells from patients with fibroadenoma (Figure 4A). As expected, stromal cells exposed to platelets in this culture condition showed increased ERK1/2 phosphorylation (Figure 4A).

Platelets are known as the major source of TGF-β in circulation; but the source of TGF-β bioavailable to tumor cells at the site of metastatic seeding remains unknown. Therefore, we subsequently sought to detect TGF-β in extracted fibroadenoma cells and platelets. A gradual and increasing detection of TGF-β was observed in the stromal fibroadenoma cells in all studied PRP concentrations. These results showed the drastic difference in Src-FAK and ERK signaling when cells were supplemented with FBS or PRP. Contrary to what is observed in fibroadenoma, stromal cells from phyllodes tumor, a rare entity analogous to fibroadenomas biphasic neoplasms, present slightly FAK Y397 phosphorylation and higher Src-FAKY925 phosphorylation than fibroadenoma cells (Figure 4B); this FAK phosphorylation at Y925 could facilitate an angiogenic response in mammary tumors [41]. Consistent with the fact that the estrogen (E2) and progesterone (P4) pathways are overstimulated in these benign tumors, we observed that phyllodes stromal cells supplemented with PRP and treated with the E2+P4 combination (100 nmoles/L) induced EMT reversion with the expression of E-cadherin by stromal phyllodes cells (Figure 4B). This was not observed with FBS supplementation in the presence of hormonal treatment (data not shown) indicating that the microenvironment is pivotal for triggering some phenotype alterations and confirming that phyllodes tumors interact locally with the microenvironment and can undergo malignancy progression from the sarcoma phenotype to metastasis [13, 35].

Using the same conditions, we subsequently investigated whether platelets and fibrin bundles could have a direct impact on the behavior of breast epithelial tumor cells using primary cells from patients with luminal A and B, and HER2+ breast tumor subtypes. Luminal breast cancers, which often include ER+ tumors, are the most prevalent form of breast tumors. However, luminal A differs from luminal B subtype by presenting good prognosis and low malignancy, and by responding to hormonal treatment [36]. Our results with the PRP supplementation in the cell medium demonstrated a clear difference between luminal A and B, results that are consistent with the characteristics of luminal A and B subtypes. We detected the EMT process in luminal A cells in the presence of the highest PRP supplementation (10%), however, the prevalence the E-cadherin expression confirmed the epithelial phenotype even when we treated luminal A cells with the hormone combination (E2+P4) (Figure 4C). N-cadherin was slightly detected in the 10% PRP supplementation in presence of E2+P4 hormones, which states the responsiveness of these tumors to hormone therapy [36]. The cascade of TGF-β/Smad2 and Snail related to EMT was investigated and similar to what was observed in N-cadherin, this cascade was significantly detected in the presence of the highest PRP concentration, and prominently detected in luminal A cells adhered to the network of fibrin bundles (Figure 4C). This cross-talk between the luminal A epithelial cells and PRP-fibrin bundles showed Smad2 expression in human platelets [37]. It is important to note that luminal A cells changed their adhesion site only when exposed to the 7.5% PRP supplementation, contrary to what was observed in the epithelial cells from luminal B and HER2+ tumors. Luminal B cells exposed to 5.0% PRP and HER2+ epithelial cells exposed to 2.5% PRP supplementation showed a fast onset of migration to early fibrin bundles. The luminal B and HER2+ breast tumor cells from the patient with the highest Ki67 positive result, a result associated with the most malignant features in breast tumor luminal B and HER2+ subtypes, showed a clear EMT induction by the PRP supplementation, with N-cadherin upregulated and E-cadherin downregulated (Figure 4D and 4E). The Src/FAK Y925 complex was clearly phosphorylated when the cell culture was supplemented with PRP. The PRP supplementation provided a fibrin matrix as important scaffolding that helped these recruited mesenchymal-like phenotype cells to attach via integrins and start migrating. These results led us to investigate the β1 and β5 integrins in breast carcinoma cells because these integrins are involved in FAK and Src activation in HER2/ErbB2-overexpressing mammary epithelial cells [38, 39]. We investigated the α6 and αv subunits where fibrin can be bound and lead to the formation of tumor cell–fibrin–platelet aggregates [39]. In both epithelial cells from breast tumor luminal B and HER2+ subtypes, alterations in the interaction of tumor cells with integrin subunits were observed in the PRP supplementation; the participation of integrin subunits α6β1 was clear when these cells were supplemented with FBS (Figure 4D and 4E). However, a significant participation of the αvβ5 integrin subunit was observed when PRP supplementation was gradually introduced, with the most significant participation being observed with 10% PRP. This links the formation of a signaling complex containing FAK and αvβ5 integrin, in a Src-dependent manner, as essential to VEGF-stimulated angiogenesis [39, 40]. Other integrins’ subunits were investigated (data not shown); however, the α6β1 integrin was predominant in the FBS supplementation and became the focus of our experiment.

Considering that prolonged exposure to TGF-β promotes EMT in many cancer cell lines [41], we investigated whether platelet-derived TGF-β could activate TGF-β/Smad and Snail signaling in breast tumor cells from patients with luminal A and B and HER2+ subtypes. We detected increased TGF-β levels in the highest PRP supplementation in luminal A and B epithelial cells, and HER2+ tumor cells. ERBB2 was detected as a consequence of TGF-β exposure (Figure 4C–4E).

In cases of tumors that overexpress ERBB2, such as HER2+, the PRP supplementation further increased the already overexpressed ERBB2 (Figure 4E).

The main result from our data was the fact that luminal B and HER2+ cells attached to the network of fibrin bundles and expressed EMT markers such as Smad2 and Snail, markers that can trigger the transition of cells into more mobile types that spread regardless of the presence of normal biological controls that restrict metastasis (Figure 4D and 4E).

We investigated whether platelet-derived TGF-β could activate TGF-β/Smad signaling in tumor cells. We found increased levels of active and latent TGF-β1 in the medium derived from the co-culture of tumor cells and 5% PRP, after the PRP was removed (Figure 4F). We have strong indications that PRP-induced EMT is dependent on TGF-β signaling. Interestingly, adding a TGF-βRI inhibitor (SB431542) or a TGF-β1 blocking antibody abolished PRP-induced EMT in Luminal B and HER2+ tumor cells (Supplementary Figure 2).

### Breast cancer cells under PRP supplementation efficiently induce endothelial cell tube formation

Angiogenesis assays to assess pro-angiogenic factors were used *in vitro* to correlate the pathway, activated in different subtypes of breast cancer cells under PRP supplementation, to the angiogenesis process (Figure 5). Therefore, we analyzed the ability of stromal and epithelial breast tumor cells, in the presence of platelets and fibrin resulting from PRP supplementation, to stimulate angiogenesis using a co-culture model with human umbilical vein endothelial cells (HUVEC). In addition, we showed that the gel-like structure resulting from PRP supplementation detected in our cultures mimics the tumor microenvironment matrix. We initially observed that stromal cells from fibroadenoma samples were able to stimulate minimally cluttered capillary tube formation (Figure 5A). In a significant contrast, stromal cells from phyllodes samples showed approximately 4-fold enhanced angiogenesis-promoting ability (Figure 5B and 5F). We then noted that epithelial cells from luminal A and B and HER2+ breast tumor subtypes induced capillary tube formation. Luminal A epithelial cells promoted a slight formation of angiogenic structures in human endothelial cells (HUVECs) compare with luminal B and HER2+ cells. After 16 h of coculture, the capillary tube structure was still intact and well defined (Figure 5C). Moreover, luminal B and HER2+ epithelial cells present 6.5-fold higher angiogenic structures than luminal A epithelial cells. The capillary tube structure was stable and defined; it remained a robust, defined, and bona fide angiogenic structure in both luminal B and HER2+ cells even after 16 h of coculture (Figure 5D–5F). In addition, endothelial cells were still viable after 24 and 36 h of coculture with PRP supplementation (data not shown), unlike other cells in standard *in vitro* HUVEC assays, which are already apoptotic after 24 h of angiogenesis assay [41]. Because the control breast tumor cells under FBS supplementation were unable to stimulate angiogenesis in all breast cancer subtypes, the importance of a coating that mimics ECM, such as Matrigel, is reinforced.

**Figure 6:**
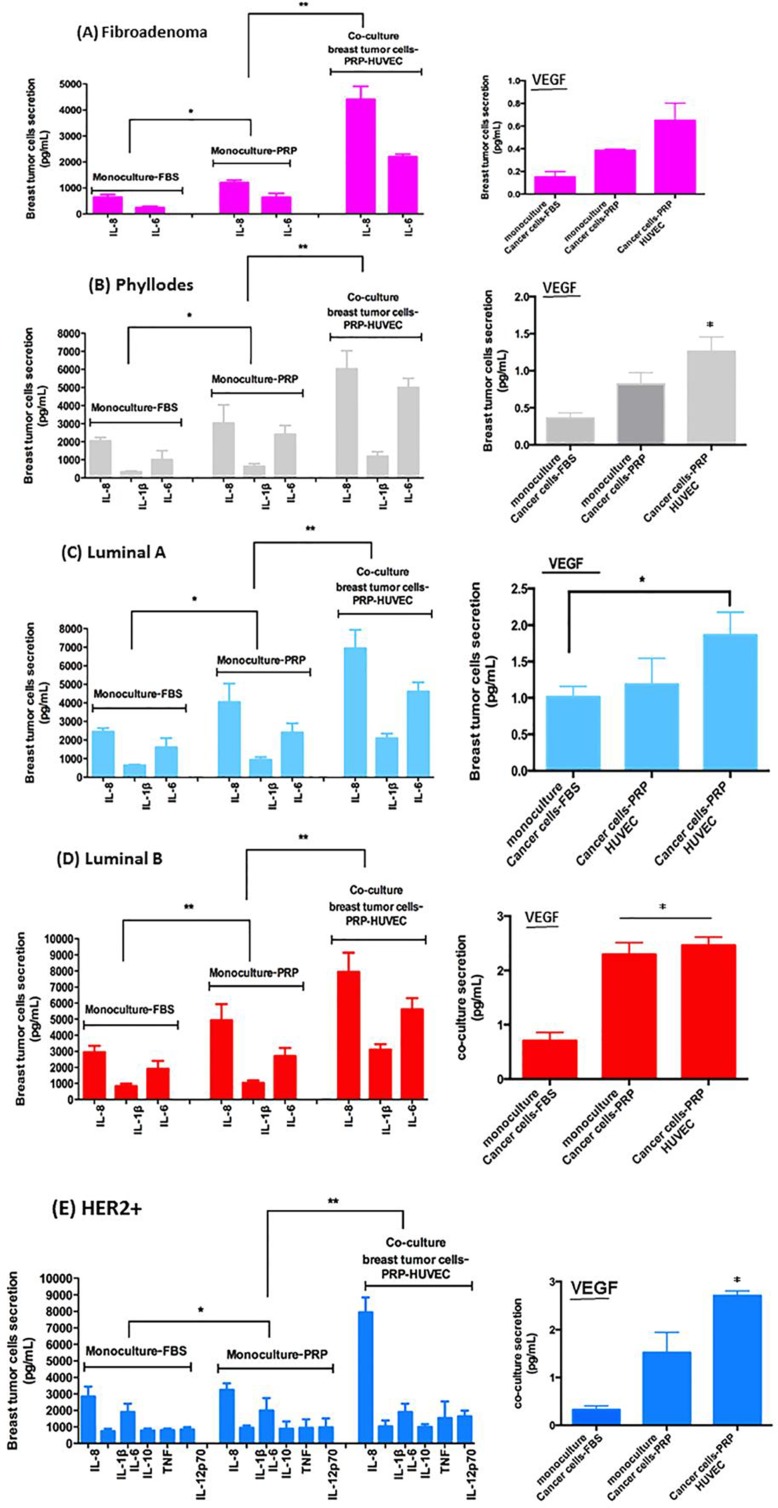
Relative levels of indicated cytokines in CM of co-culture of breast tumor cells under PRP supplementation and HUVEC (**A**–**E**) The conditioned medium was recovered to measure protein secretion by flow cytometer in co-cultured cells in pg/ml). (**F**–**J**) VEGF concentration in CM after 8–16-hour culture measured by flow cytometry (human VEGF flex set’, BDTM cytometric bead array, CBA, BD Biosciences, USA). Values are the means ± SEM of duplicate determinations in co-cultures from all specimens collected from patients with fibroadenoma, phyllodes, luminal A and B, and HER2+ *p ≤ 0.05, **p ≤ 0.001.

A human cytokine array on various conditioned medium (CM) was conducted to identify proangiogenic factors carried by cross-talking between breast cancer cells-PRP-HUVEC. When we compared CM of stromal cells from fibroadenoma and phyllodes tumors, a number of proangiogenic and proinflammatory human cytokines showed significantly higher concentration in CM from phyllodes stromal cells than fibroadenoma cells, including interleukin-6 (IL-6), IL-8, and vascular endothelial growth factor (VEGF), [IL-6 (*p* = 0.044), IL-8 (*p* = 0.012), and VEGF (*p* = 0.05)] (Figure 6A and 6B).

We detected CM differences between epithelial cells from luminal A and B breast cancer subtypes under co-culture after 8–16-hour culture measured by flow cytometry. In sharp contrast, CM from luminal B epithelial cells presented significantly higher concentrations of pro-angiogenic and pro-inflammatory human cytokine than CM from luminal A epithelial cells. The cytokines in CM from luminal B epithelial cells included IL-6 (*p* = 0.033), IL-8 (*p* = 0.05), IL-1β (*p* = 0.05), and VEGF (*p* = 0.040) (Figure 6C and 6D). Finally, HER2+ epithelial cells showed drastic differences in cytokine types and concentrations detected in CM when compared with the luminal breast cancer subtype. These cytokines included IL-6, IL-8, IL-1β, IL-10, TNF, IL-12p70, and VEGF (all with *p* ≤ 0.05) (Figure 6C and 6E).

Homotypic cultures of one of the cell types were used as controls, and the results reinforce that cross-talking between breast cancer cells-PRP-HUVEC are primordial to VEGF increase (data not shown).

**Figure 7:**
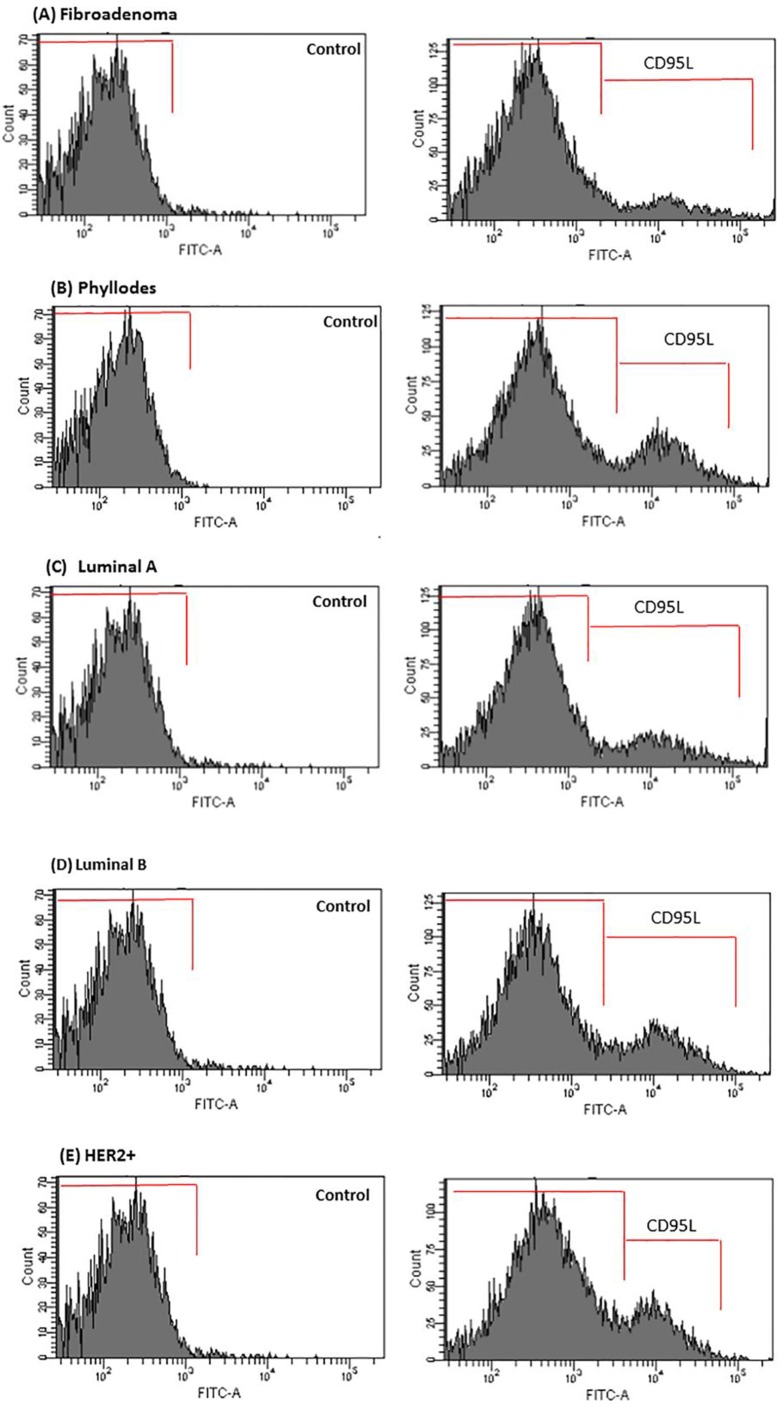
CD95L secretion is enhanced in CM of co-cultured breast tumor cells under PRP supplementation-HUVEC (**A**–**E**) After 16 h, media were removed and analyzed for CD95L by flow cytometry in the Accuri C6 (BD Biosciences, USA). Values are the means ± SEM of duplicate determinations in co-cultures from all specimens collected from patients with fibroadenoma, phyllodes, luminal A and B, and HER2+*p ≤ 0.05.

We examined the activation status of CD95 L in order to understand whether some of these cytokines were functioning at responding tumor sites. CD95 L plays an important tumor supportive role in both breast tumor cells and endothelial cells controlling the angiogenic process. CD95 L (soluble), or Fas-L is an effector regulator of endothelial cell survival in recently formed endothelial cells related with the cytokines identified in our screen, including IL-8 and VEGF [42, 43]. Using an antibody specific to the CD95 L soluble form, we observed that the levels of CD95 L in the flow cytometry analysis were negligible in the FBS and PPP supplemented control breast tumors cells (data not shown). In addition, CD95 L was not detected in stromal cells from fibroadenoma biopsies (Figure 7A). Conversely, stromal cells from phyllodes biopsies showed a slight presence of CD95 L in CM of co-cultures with stromal phyllodes cells-PRP-HUVEC (Figure 7B). In marked contrast, CD95 L staining was abundant in responding tumors that presented ER+/PR+ and HER2+/Erbb2 expression, such as luminal A and B and HER2+ subtypes (Figure 7C–7E).

Taken together, these data established that PRP supplemented breast tumor cells induced the formation of a gel-like structure that mimics the tumor microenvironment matrix. Furthermore, the presence of viable platelets transmits paracrine signals to both breast tumor cells and HUVEC cells to induce capillary tube formation through cytokine pathways. This process was tumor-subtype specific, occurring mainly in luminal B and Her2+ breast cancer subtypes. Therefore, these findings underscore the importance of the complete complement of cytokines cargo carried by platelets under different pathological conditions such as in breast cancer subtypes.

## DISCUSSION

The tumor-stromal interface is known to co-evolve with epithelial cancer from preinvasive to malignant stages [2, 41]. The mechanism of interface interactions in driving tumor progression and defining the differences between the so-called reactive stroma of breast tumors and normal stroma in terms of their influence on tumor cell migratory/invasive properties is largely unknown. Thus, considering the importance of the tumor microenvironment and the necessity to mimic the structurally constrained tumor-stromal interactions *in vivo*, we developed a culture method that is devoid of animal-derived products such as fetal bovine serum (FBS). Therefore, we used PRP supplementation as an alternative to FBS in primary cultures of breast tumor cells from biopsies from women with different breast cancer subtypes. PRP contains several growth factors, including a high level of TGF-β1 [29]. In the present study, PRP was used not only for cell supplementation but also for providing tumor cell support by mimicking the tumor microenvironment matrix with the network of fibrin bundles. It has generally been accepted that a network of fibrin bundles is formed at the site of injury and provides the temporary matrix, a solid substrate for stromal and transformed epithelial cells [18]. In addition, it acts to support the initial endothelial cell response needed for the angiogenic process [18, 39, 40]. Based on these established concepts, our observation shows that at 30 min of PRP supplementation, the liquid culture medium was converted into a gel-like structure that acts as a solid substrate which is tumor-subtype and cancer cell-type specific. PRP at 2.5% is the minimal concentration required for this gel formation. This implies that breast tumor cells with increased malignancy properties, in our case, stromal cells from phyllodes benign breast tumor and epithelial cells from luminal B and HER2+ breast cancer subtypes changed the FAK Tyr residue phosphorylation from FAKY397 to FAKY925. This finding shows the alterations on the focal adhesion complex in the presence of PRP, contrary to what is observed using FBS supplementation. Therefore, FAKY925 cooperates with Src disrupting E-cadherin-based intercellular cadherin junctions and promoting E-cadherin internalization during cancer progression [44–47], and hence, facilitating EMT and enhancing tumor cell motility and invasiveness. It has been shown that TGF-β-induced EMT requires Src or integrin-dependent FAK activation that leads to E-cadherin downregulation [48, 49]. Our data show the expression inversion of integrin subunits with increasing PRP supplementation in luminal B and HER2+ breast tumor cell culture: the expression of α6β1 subunits decreases and that of αvβ5 subunits increases according to the formation of tumor cell–fibrin–platelet aggregates [39].

As mentioned, our PRP preparations contained high levels of viable platelets (TGF-β and VEGF source) and low concentrations of fibrinogen (fibrin precursor); in addition, our primary cultured breast tumor cells produced thrombin at detectable levels. Furthermore, other proteases were detected in CM from stromal and epithelial breast tumor cells and are possibly involved in the conversion of fibrinogen to fibrin that takes place in the *in vitro* system. In fact, thrombin and other serine proteases from plasma and platelets contribute to the formation of fibrin bundles, however, in the presence of PPP, breast tumor cells cannot effectively contribute to the formation of solid substrates. This finding reinforces the fact that platelets are important as a thrombin source and responsible for stimulating breast tumor cells to express different proteases [4, 18, 40, 50]. The epithelial cells from luminal B and HER2+ breast cancer subtypes show the fastest formation of the network of fibrin bundles and display the highest number of cells migrating from the plastic surface to this network. According to our results, PRP supplementation stimulates fibroblasts from phyllodes benign breast tumors in the presence of steroid hormones to revert from mesenchymal to the epithelial phenotype. This phenotypic change reflects an altered breast tumor cell behavior and, thus, can be a critical step in cell survival at metastatic sites [51]. In contrast, the PRP supplementation promotes EMT in luminal B and HER2+ epithelial breast cancer cells with the expression of metastasis markers such as Smad2 and Snail1 due to the TGF-β1 mediated pathway [34]. These results corroborate those from other studies showing that the binding of TGF-β to receptors causes the phosphorylation of cytoplasmic Smad proteins and their dispatch to the nucleus activating Snail, which induces invasive behavior in malignant breast tumor cells [52].

Another important finding is that the co-culture of stromal and epithelial cells from the same breast cancer subtype, and the same tissue sample, triggers paracrine signals between stromal cells and epithelial cells, which is revealed by increased MMP-2 and MMP-9 secretion following exposure to PRP (platelets) supplementation. These results suggest a higher capacity to degrade the extracellular matrix and invade the surrounding environment compared to monolayer cell culture under FBS supplementation, as observed in luminal B and HER2+ breast cancer subtypes.

It has long been thought that certain tumors must undergo an angiogenic switch in order to break dormancy [53]. Thus, it has become increasingly clear and accepted by researchers that platelets provide a rich source of proangiogenic factors in opposition to the changing perspectives of tumors intrinsically acquiring the angiogenic potential to angiogenesis considered as the consequence of processes that are initiated at distant sites. Recent studies established that paraneoplastic thrombocytosis is associated with reduced patient's survival [1, 54, 55], indicating that platelets might not be just an epiphenomenon of malignancy but actual drivers of malignancy progression.

Our results using PRP supplementation and primary human breast tumor specimens is supported by the following clinical observations: i) fibroadenoma shows particularly benign evolution; ii) Phyllodes tumors can undergo malignant progression to metastasize; iii) Luminal breast cancers, which often include ER+ tumors, are differentiated and associated with good prognosis and patients’ response to current treatment therapies; iv) Endocrine treatment (luminal A subtype) being luminal B and tending to high malignancy progression and metastasis; v) HER2+ subtype being a metastatic breast cancer with poor prognosis [14, 15].

In primary carcinomas, secreted growth factors and cytokines contribute to inducing EMT in the microenvironment of tumor cells, and stimulating the angiogenic process. Clearly, the crosstalk between epithelial cells and components of the tumor stroma is distorted in cancer to promote tumor growth and progression. Thus, the difference between breast cancer subtypes was not restricted to malignancy progression, but also dependent on the group of players that induced the malignancy progression. Our study indicates that the range of cytokines carried by breast tumor cells (stromal and epithelial cells) under PRP supplementation and in co-culture with HUVEC was tumor-subtype and cancer cell-type specific; these cytokines include IL-8, IL-6, TNF-α, and VEGF but are not limited to these ones. These cytokines mediate pro-inflammatory and pro-angiogenic responses in the responding tumor microenvironment to promote tumor progression. Among the cytokines produced by breast cancer cells, the role of TGF-β in tumor development and progression has been extensively studied [1, 4, 34, 52, 55]. Acting as a tumor suppressor early on, TGF-β later becomes a key factor in promoting tumor progression, metastases, and resistance to treatment [56]. Therefore, targeting these other factors or the platelets that carry them in appropriate cohorts of patients should open new therapeutic windows.

HER2+ showed the most peculiar cytokine profile with the expression and production of the pro-inflammatory cytokines IL-8, IL-6, IL-10, IL-1β, and IL-12 p70, but not IL-12 p40. These findings are contrary to those described by [57]. There is no consensus on whether IL-12 p70 is involved in breast cancer progression in relation to our results because the Heckel study was conducted with FBS supplementation. Nevertheless, some cytokines were found to be significantly elevated in sera of metastatic breast cancer patients with HER2-positive cancers compared with HER2-negative cancers [57]. It is important to note that there are factors that are different from those provided by FBS in the stroma microenvironment matrix [58, 59].

The control on the formation of new vessels is stimulated by resting endothelial cells with angiogenic agents such as VEGF, which creates activated growing endothelial cells that soon express the Fas death receptor on their surface. Subsequently, mature and recently formed endothelial cells are stimulated to secrete CD95 soluble or FasL, the ligand of Fas. However, because only activated endothelial cells display the Fas receptor, they are preferentially induced to enter apoptosis while FasL, secreted by mature and resting endothelial cells, cannot be induced to enter apoptosis because they cannot bind and respond to secreted FasL. In this scenario, our data shows increased soluble CD95 (FasL) in the CM co-culture with luminal B and HER2+ epithelial breast tumor cells and HUVEC, which are tumor cells that have enhanced formation of the capillary tube structure. This seems to explain that VEGF (angiogenic factor) expression increased by breast tumor cells was stimulated by the Fas receptor (cell death receptor) expression in HUVEC as a control of vessel formation in active proliferation [60].

## MATERIALS AND METHODS

### Chemicals and biochemicals

All chemical reagents were of analytical grade. Deionized and ultra-filtered water from the Milli-Q ultrafiltration system was used. The biochemical assays were conducted using commercially available kits. Dulbecco's Modified Eagle's Medium (DMEM/F12), Roswell Park Memorial Institute Medium (RPMI 1640), and phosphate buffered saline (PBS), insulin, epidermal growth factor, hydrocortisone, cholera toxin, 3-(4,5 dimethylthiazol-2-yl)-2,5-diphenyltetrazolium bromide (MTT), CA074 (cathepsin B inhibitor), phosphatase inhibitor cocktail (sodium orthovanadate), and protease inhibitor cocktail [104 mM 4-(2-aminoethyl) benzenesulfonyl fluoride (AEBSF), 80 μM aprotinin, 4 mM bestatin, 1.4 mM E-64, 2 mM leupeptin and 1.5 mM pepstatin A], and TGF-βRI inhibitor (SB431542) were purchased from Sigma (St. Louis, MO, USA). Fetal bovine serum (FBS), penicillin, and streptomycin were obtained from Gibco^®^ (Gaithersburg, USA). Trypsin 2.5% (v/v) was purchased from Cultilab (São Paulo, Brazil). Anti-phospho and total Src-Tyr-416, FAK-Tyr-397, FAK-Try-925, E-cadherin, N-cadherin, anti-integrin α_6_, β_1_, α_v_, β_5_ rabbit antibody, TGF-β, Smad2 and Snail monoclonal antibodies, and β-actin and IgG conjugated with horseradish peroxidase from rabbit or mouse were obtained from Cell Signaling Technology (Danvers, USA). Anti-EGFR mouse antibody was purchased from Santa Cruz Biotechnology (Dallas, Texas, USA). Anti-TGFb1 blocking antibody was from R&D systems (Minneapolis, USA). The Benzoyl-Phe-Val-Arg-MCA substrate and dithiothreitol (DTT) were from Calbiochem (Darmstadt, Germany). Fluorescein isothiocyanate (FITC)-labeled anti-cytokeratin 18, E-cadherin, N-cadherin, Alexa Fluor^®^ 594-labeled anti-vimentin, phalloidin, and anti-cytokeratin 18 antibodies were obtained from BD Biosciences (California, USA); the super signal^®^ west pico chemiluminescent substrate and micro BCA^™^ were purchased from Thermo Scientific (Rockford, Illinois, USA). The MitoTracker Orange (MTO) and LysoTracker Green were obtained from Molecular Probes (Oregon, USA).

### Collection of platelet-rich plasma (PRP)

Platelet-rich plasma (PRP) was obtained from donors from the Charitable Association of Blood Collection – COLSAN in São Paulo, SP, Brazil. This part of the study was carried out in accordance with the Declaration of Helsinki and approved by the Institutional Ethics Review Board (CEP1917/11) from the Federal University of São Paulo (UNIFESP). Patients who volunteered to participate in the study signed a written informed consent form prior to participation.

### Clinical specimens and sample collection

This part of the study was approved by the Ethics Committee of research of the Federal University of São Paulo (CEP0858/10). Patients who volunteered to participate in the study signed a written informed consent form prior to participation.

Mammary tissues were obtained from patients in the Mastology Discipline/Department of Gynecology at the Federal University of São Paulo/São Paulo Hospital (UNIFESP/HSP). Women undergoing diagnostic biopsies or total or partial mastectomies were recruited. Primary mammary cell culture was prepared from biopsies obtained from 21 women, aged 34 to 67, with different subtypes of breast cancer. The collected mammary tissue was separated into two parts: one for histological analysis according to the previously criteria [61], and the other for cell culture. Data on the diagnosis of patients’ breast tumor subtype, hormonal status, and histological grade were recorded (Table 1).

### Tissue isolation and primary cell cultures

Primary mammary cell culture was prepared from 21 biopsies from women with different subtypes of breast cancer (Table 1). Tissue samples were rinsed, minced into small pieces, and digested with collagenase IA (0.05 mg/ml) for 16 h under constant stirring at 37°C. The next day, epithelial cells were separated from stromal cells using the 3-step differential centrifugation method based on a modification of the procedure described by [62]. The collagenase digest was centrifuged at 40 g for 1 min; the pellet, which contained organoids, was retained and the supernatant centrifuged at 100 g for 2 min to pellet epithelial cells. The epithelial cell pellet was retained and the supernatant centrifuged at 200 g for 4 min to pellet stromal and red blood cells. All three pellets were washed twice with PBS containing 1% of penicillin and streptomycin, and subsequently plated in selective media. Cells from organoid, epithelial, and stromal preparations were placed in primary flasks (Becton–Dickinson Labware, Le Pont de Claix, France) and cultured in two types of medium. Epithelial cells were cultured in Dulbecco's modified Eagles’ medium (DMEM)/F-12 without phenol red, with 10% fetal bovine serum (FBS), 1% penicillin/streptomycin containing 10 μg/mL insulin, 20 ng/mL epidermal growth factor, 0.5 μg/mL hydrocortisone, and 100 ng/mL cholera toxin. Fibroblasts were grown in DMEM/F-12 without phenol red, with 10% FBS, and 1% penicillin/streptomycin; cells were maintained in a humidified incubator at 37°C and 5% CO_2_. After 24 h incubation, non-adherent cells were washed out, and adherent epithelial and stromal cells were characterized by immune staining with FITC-labeled anti-cytokeratin 18, E-cadherin, N-cadherin, Alexa Fluor^®^ 594-labeled anti-vimentin, phalloidin, and anti-cytokeratin 18 antibodies.

### Preparation of PRP as supplement

Pooled venous blood samples (type AB+) from 20 healthy and non-smoking donors, aged 24 to 45 years (10 females and 10 males with average age of 31.5 ± 7.1) were used to obtain platelet-rich plasma (PRP), which was prepared as previously described [63] with minor modifications. Briefly, pooled venous blood was collected into plastic tubes without anticoagulant; PRP was obtained by centrifugation at 141 ×g for 12 min at room temperature. Platelet counts were obtained on KX-21N System (Illinois, USA) with an average of 290 (× 10^3^/μL) and range between 241 and 395 (× 10^3^/μL). The pooled PRP was bottled, cell culture tested for mycoplasma and endotoxin, and immediately used as cell supplement. PRP lacks antibodies against A and B blood type antigens. Therefore, sera containing any antibodies that could react with the cultured cells, not just anti-ABO antibodies, should be avoided, especially if the cells are destined to be used for therapy. The conditioned medium (CM) was prepared after the PRP preparation; primary cells were seeded in six-well plates (5 × 10^4^ per well) and incubated at 37°C in a humidified atmosphere with 5% CO_2_. After 24 h incubation, non-adherent cells were washed out. Conditioning of epithelial and fibroblast cells began by using PRP replacing FBS in the following conditions in each well: 10% FBS and no PRP (control); 7.5% FBS and 2.5% PRP; 5% FBS and 5% PRP; 2.5% FBS and 7.5% PRP; and 10% PRP and no FBS. PPP (platelet-poor plasma) was used as negative control. These conditions allowed the network of fibrin bundles to be formed in cell culture as a solid substrate and allowed the transition of cells from focal contacts in the plastic surface to the fibrin bundle over the cultured cells. After 2 and 16 h of culture, the morphology of cells was microscopically evaluated and photographed to determine alterations. These cells were subsequently harvested for western blot and flow cytometry analysis. Co-culture experiments were carried out with primary cells at passage 2–3 with the intent of accumulating large amounts of cells while avoiding cell-aging effects. In some cases, the epithelial tumor cell cultures from patients with the luminal breast cancer subtype were treated with the steroid hormones 17 β-estradiol (E_2_) and/or progesterone (P_4_) at 100 nmol/L because these subtypes of breast cancer are more responsive to hormonal therapy [64].

### Organotypic culture – 2D cultures/co-cultures

Primary epithelial and stromal cells from women with different subtypes of breast cancer (Table 1) were directly co-cultured for western blot and zymography analyzes, with and without HUVEC (human umbilical vein endothelial cells, kindly provided by Prof. Júlio Scharfstein from Rio de Janeiro, Brazil and used between passages 3 and 6) in the presence of PRP supplementation. Three different cell culture conditions were evaluated. In the first condition, epithelial cells were plated at 4 × 10^4^ density in six-well microplates (Corning Inc.) with HUVEC. The medium was removed after the cells’ attachment and replaced with six different cell culture media: DMEN/F12 prepared at 10% FBS and no PRP (control); 7.5% FBS and 2.5% PRP; 5% FBS and 5% PRP; 2.5% FBS and 7.5% PRP; and 10% PRP and no FBS in each well. HUVEC was plated 1:1 with each cell type after PRP supplementation. Cells were harvested for western blot analysis after 2 and 16 h of culture. In the second cell culture condition, stromal cells were co-cultured with HUVEC in six different media as described above. Cells were harvested after 2 and 16 h of culture for western blot analysis. The third condition consisted of a co-culture of stromal and epithelial cells. Stromal cells (4 × 10^4^ per well) were grown to confluence in monolayers, epithelial breast cancer cells were subsequently added to each well, and the medium was replaced with DMEN/F12 prepared as 10% FBS and no PRP (control); 7.5% FBS and 2.5% PRP; 5% FBS and 5% PRP; 2.5% FBS and 7.5% PRP; and 10% PRP and no FBS. After 16 h of culture, the medium was removed from the wells and analyzed by zymography. All primary cells from the 21 mammary biopsies were used in the three cell culture conditions. Assays were performed in triplicate.

### Detection of thrombin activity in primary breast tumor cells

Epithelial and stromal cells were seeded (10^4^ per well) into six-well plates at 70% confluence in their respective culture medium: 1) DMEN/F12 without phenol red with 10% FBS and 1% penicillin/streptomycin containing 10 μg/mL insulin, 20 ng/mL epidermal growth factor, 0.5 μg/mL hydrocortisone, and 100 ng/mL cholera toxin; and 2) DMEN/F12 without phenol red and with 10% FBS. Subsequently, the medium was changed and each well received 10% FBS and no PRP (control); 7.5% FBS and 2.5% PRP; and 5% FBS and 5% PRP. The total cell migration from the plastic surface to the network of fibrin bundles was observed at concentrations up to 2.5% PRP, which prevented the analysis of CM in the HER2+ cells. Therefore, thrombin activity for HER2+ cells was only investigated at 2.5% PRP.

After 48 h, the supernatant was removed and centrifuged (563 × g for 10 min), resulting in the conditioned medium (CM). The total protein concentration in the CM was quantified by the micro BCA kit according to the manufacturer's instructions (Pierce). The degradation of the Benzoyl-Phe-Val-Arg-MCA substrate by proteins was evaluated in the CM of epithelial and stromal cells from the studied biopsies through an enzymatic reaction composed of 50 mM Tris/HCl buffer pH 7.5 containing 0.05 M CaCl_2_, 100 μg of total protein present in the conditioned medium, and 0.4 mM Benzoyl-Phe-Val-Arg-MCA substrate. After 16 h at 37°C, fluorescence was measured on a FluoroCount Packard^TM^ (SpectraCount model; Packard) spectrofluorometer set at 355 nm excitation and 460 nm emission. Thrombin activity was assessed in the cells extracted from the 21 breast biopsies.

### Combinatorial libraries of internally quenched fluorescent substrates

The fluorescence resonance energy transfer (FRET) peptide libraries Abz-GXXzXXQ-EDDnp were obtained as described elsewhere [65] through the solid-phase peptide synthesis strategy as previously described [66, 67]. An automated bench-top simultaneous multiple solid-phase peptide synthesizer (PSSM 8 system, Shimadzu, Japan) was used to synthesize peptides using the Fmoc-procedure. Stock solutions of peptides were prepared in DMSO, and the concentration measured spectrophotometrically using the molar extinction coefficient of 17.300 M-1cm-1 at 365 nm.

The library was used in the screening of endopeptidase activities in the same conditions used for the detection of thrombin activity. Metalloendopeptidase and serine proteinase activities were characterized using the fluorogenic peptide Abz-GFSPFRQ-EDDnp [65].

### Hydrolysis of FRET peptides

The hydrolysis of FRET peptides and libraries were quantified using a Gemini M5 microplate reader (Molecular Devices Spectramax, Sunnyvale, CA, USA) by measuring fluorescence at 420 nm following excitation at 320 nm. The concentrations of protein in the total CM (final concentration: 10 μg/mL) were chosen based on less than 5% of the substrate being hydrolyzed over the course of the assay. The DMSO concentration in assay buffers was kept below 1%. Data were collected at least in duplicate in all assays. The scissile bond of hydrolyzed peptides was identified through the isolation of fragments using analytical HPLC followed by the determination of their molecular masses by LC/MS using an LCMS-2010 equipped with an ESI-probe (Shimadzu, Japan).

### Proteinase inhibition assays

The CM from breast tumor cells was pre-incubated with each inhibitor for 30 min at 25°C prior to the addition of substrates in the proteinase inhibition assays. The classic protease Inhibitors used were: metalloprotease inhibitors –2 mM ο-phe (ο-phenantroline) and 5 mM EDTA (ethylene diamine tetra acetic acid); serine protease inhibitors - 100 μM TLCK (tosyl-L-lysyl-chloromethane hydrochloride, trypsin-like serine protease inhibitor), 100 μM TPCK (tosyl phenylalanyl chloromethyl ketone, chymotrypsin-like serine protease inhibitor); and cysteine protease inhibitors −5 μM E64 (irreversible inhibitor of cysteine-proteases) and 1 μM CA074 (irreversible inhibitor of cathepsin B (cat B)).

### Cysteine protease activities

The activity of cysteine proteases was quantified through fluorometric assays using either carbobenzoxy-Phe-Arg-7-amide-4-methylcoumarin (Z-FR-MCA, Sigma-Aldrich Corp., St. Louis, MO, USA) or ε-NH2-caproyl-Cys(Bzl)-Cys(Bzl)-MCA (synthesized by Prof. Dr. Maria A. Juliano) [68] as substrates. These substrates were used to quantify total cysteine proteases and cat B, respectively. Incubations were carried out in black microplates (Corning, MA, USA) in 50 mM phosphate buffer at pH 6.3 containing 10 mM EDTA. The enzymes (100 μg of protein) were pre-activated by incubating conditioned medium aliquots with 2 mM dithiothreitol (DTT, 10 min at room temperature) and subsequent addition of the substrate (20 μM in 200 μl final volume). The fluorescence produced upon the hydrolysis of substrates was measured every 20 seconds in the FlexStation 3 (Molecular Devices, CA, USA) using λ excitation = 380 nm and λ emission = 460 nm. The assays were also performed in the presence of the following inhibitors: 1 mM phenylmethylsulfonyl fluoride (PMSF, an inhibitor of serine-proteases), 5 μM E64 (an irreversible inhibitor of cysteine-proteases), and 1 μM CA074 (an irreversible inhibitor of cat B).

### MTT cell viability assay

The viability of primary breast tumor cells (fibroadenoma, phyllodes, and luminal A and B) in the presence of 5.0% PRP supplementation, and HER2^+^ in the presence of 2.5% PRP, was measured using the MTT assay. Briefly, attached breast cells (epithelial and stromal cells at ≈2 × 10^3^ density) in 96-well microplates (Corning Inc.) were cultured in the appropriate medium without phenol red and supplemented with 2.5 or 5.0 % PRP for 24 h at 37°C and 5% CO_2_. These cells were treated with 0.5 mg/ml MTT solution (Sigma) in 10 mM phosphate buffered saline at pH 7.4 (PBS) and incubated for 2 h at 37°C and 5% CO_2_. After incubation, the medium was removed, and cells were washed. Formazan crystals in the cells were dissolved in DMSO and measured spectrophotometrically at 540 nm.

### *In vitro* angiogenesis assay on PRP

The adapted *in vitro* human umbilical vein endothelial cell (HUVEC) assay was used to investigate the impact of tumor epithelial cells/HUVEC and stromal cells/HUVEC interactions with the formation of capillary tubes on the stimulation of angiogenesis. Briefly, epithelial tumor cells and stromal fibroblasts from patients with different subtypes of breast cancer were cultured in the presence of 5.0% PRP in 6-well plates (TPP) and incubated at 37°C for 2 h to induce polymerization (as described above). HUVECs in RPMI medium supplemented with 0.2% FBS were seeded on gel-polymerized PRP in the presence of epithelial tumor cells or stromal fibroblast cells at the density of 4 × 10^4^ cells/well, and incubated at 37°C and 5% CO_2_. After 2 and 16 h of coculture incubation, cells were observed under a light microscope and photographed for subsequent counting. Homotypic cultures of one of the cell types were used as controls.

### Immunofluorescence by confocal microscopy analysis

Primary cells (epithelial, stromal, and epithelial-mesenchymal-like transition phenotype) were grown on 12-mm diameter glass coverslips on 24-well cluster plates for 2 days prior to use. The plates were pretreated with polylysine (0.01 mg/mL) before cells were plated. Cells (1 × 10^4^) were kept at 20°C in each medium before being mounted on microscope slides and examined in *in vitro* confocal microscopy at 37°C.

Cells were fixed with 2% paraformaldehyde/PBS for 30 min, washed three times with 0.1 M glycine/PBS, and permeabilized with 0.01% saponin/PBS for 15 min to verify the localization of cytokeratin-18, vimentin, and phalloidin-cytoskeleton. All markers were analyzed by incubation with mouse anti-protein and rabbit anti-protein (1:250) primary antibodies for 1 hour. Subsequently, cells were washed three times with PBS and incubated with secondary antibodies conjugated with anti-mouse and anti-rabbit IgG FITC 488 and Alexa Fluor^®^ 594 (1:250), respectively, for 1 hour in the dark. Nuclei were stained with DAPI (4′,6-diamidino-2-phenylindol; Invitrogen; 20 μg/ml) for 15 min. Coverslips were mounted on microscope slides with Fluoromont G (Immunkemi, Stockholm, Sweden) for analysis by confocal microscopy.

The impact of PRP supplementation on metabolic cell levels, mitochondria, and lysosome quantification were investigated in epithelial and stromal breast tumor cells. Mitochondria and lysosomes were labelled with MitoTracker Orange (MTO) (Molecular Probes) and LysoTracker Green (1:500) (Molecular Probes), respectively, for 20 min at 37°C in the dark, and fluorescent signals were recorded with a confocal laser scanning microscope (Zeiss LSM510 META, Heidelberg, Germany) and Plan-Neofluor ×40 and ×63 oil-immersion 1.3 NA lenses with excitation filters at 488 nm (green) and 551 nm (red) with laser-line argon/krypton. The pinhole device was adjusted to capture the fluorescence of one airy unit. The images were processed using the LSM 510 (Zeiss) and Image J (NIH, Bethesda, MD). Immunofluorescence confirmed using LeicaMCL7000 system.

### Protein preparation and Western blot analysis

Protein extraction and western blot analysis were performed according to standard procedures. Briefly, total protein was isolated from the control and experimental cells using an ice-cold lysis buffer (20 mM Tris, 300 mM NaCl, 2 mM EGTA, and 2% NP-40 (nonidet-P40)) at pH 7.5 containing the Roche Complete Protease Inhibitor Cocktail (Basel, Switzerland), phosphatase inhibitors, 1 mM Na_3_VO_4_ (sodium orthovanadate), and 100 mM NaF (sodium fluoride). These extracts were stored at −80°C until analysis. The total protein content was measured using the Micro BCA Protein Assay kit from Pierce (Rockford, IL, USA). Total proteins in the cell lysate (80 μg, standardized for all cellular samples) were separated by electrophoresis under denaturing and non-reducing conditions (SDS-PAGE) using 5% stacking and 10% separating gels. Proteins were transferred to GE Healthcare PVDF (polyvinylidene difluoride) membranes (Pittsburg, PA, USA) through 2.4-h of electroblotting at 200-mA constant current in blotting buffer (20 mM Tris base, 150 mM glycine, and 20% methanol) using the Mini Trans-Blot Electrophoretic Transfer Cell from Bio-Rad (Hercules, CA, USA). Membranes were quenched for 2 h with 0.1 to 1% bovine serum albumin (BSA) in TBST buffer (200 mM Tris/HCl buffer pH 8.0 containing 150 mM NaCl and 0.05% Tween 20) and incubated overnight at 4°C with anti-Src rabbit antibody (Tyr-416), anti-phospho-Src rabbit antibody, anti-FAK rabbit antibody (Tyr-397), anti-phospho-FAK rabbit antibody, anti-FAK rabbit antibody (Tyr-925), anti-phospho-FAK rabbit antibody, anti-ERK 1/2 (p42/44 MAPK) rabbit antibody, anti-phospho-ERK 1/2 (p42/44 MAPK) rabbit antibody, anti-E-cadherin rabbit antibody, anti-N-cadherin rabbit antibody, anti-Smad 2 rabbit antibody, anti-Snail rabbit antibody, anti-TGFβ1 mouse antibody, anti-integrin α_6_, β_1_, α_v_, β_5_ rabbit antibody, anti-EGFR mouse antibody, and anti-β-actin; all diluted in 1% BSA in TBST and all from Cell Signaling Technology (Beverly, MA, USA). The membranes were sequentially washed three times with TBST after this incubation. The detection of the chemiluminescent signal was performed using the ECL gel documentation system (UVITEC, Cambridge, UK) and Super Signal from Pierce (Rockford, IL, USA). The densitometry analysis was performed in the ImageJ software (NIH, USA) using the phospho-proteins/total-proteins ratio and β-actin as controls. Cells were also characterized by immune staining with FITC-labeled E-cadherin, N-cadherin, PAI-1, and claudin, and assessed using fluorescence-activated cell sorting (FACS) by Accuri C6 (BD-Biosciences, San Jose, CA, USA).

### Flow cytometer analysis - detection of cytokines through CBA, VEGF, and CD95

Cytokines were assayed using the Inflammation CBA Detection Kit (BD Biosciences). The protein levels of interleukin (IL)-8, IL-1β, IL-6, IL-10, tumor necrosis factor (TNF), and IL-12p70 were quantitatively measured in the CM from tumor epithelial cells/HUVEC and stromal cells/HUVEC interactions in the presence of 2.5 or 5.0% PRP supplementation and seeded (2 × 10^4^ per well) into 6-well tissue culture plates (TPP). Cells were cultured in a 1:1 ratio. The same conditions were used to assess VEGF concentrations in supernatant samples of culture media by flow cytometry using the Human VEGF flex set kit (BDTM Cytometric bead array, CBA, BD Biosciences, USA) according to the manufacturer's instructions. The VEGF concentrations in the media removed from wells were calculated based on a standard curve provided in the kit, ranging from negative (control) to 5,000 pg/mL. Events acquisition was performed on the fluorescence-activated cell sorter Cell surface Accuri C6 flow cytometer and CFlow plus software (Accuri Cytometer). All 21 samples were analyzed in duplicate.

The analysis of the CD95 L soluble form (APO-1) using FITC was performed on the same samples. After co-culture, CM-cells were concentrated and then incubated with 1 μg of anti-CD95 L (FasL) or Ab isotype control in 2% BSA/PBS on ice for 30 min according to the manufacturer's instructions. CD95 L staining was measured by flow cytometry analysis (Accuri C6, BD Bioscience).

### TGF-β1 ELISA

TGF-β1 levels were detected in tissue-culture-conditioned medium (8–16 hr), platelet-rich plasma either by direct assay (active TGFβ1), or following acid treatment to activate latent TGFβ1 (total TGFβ1) with the Quantikine TGFβ1 immunoassay kit (R&D Systems).

### Zymography

Conditioned medium with 2.5% PRP supplementation from homotypic cultures and co-cultures of primary cells (epithelial and stromal cells as the feeder layer) were collected and centrifuged to remove cellular debris. Volumes of conditioned media normalized to the number of cells (with total protein 10 to 20 μg) were mixed with buffer and loaded onto a 7.5% acrylamide/bisacrylamide separating gel containing 0.02% (w/v) gelatin. After electrophoresis, the gel was incubated in 2.5% Triton X-100, rinsed in distilled water, and incubated for 16 h at 37°C in buffer containing 50 mM Tris pH 7.6, 20 mM NaCl, 5 mM CaCl_2_, and 2 μM ZnCl_2_. The gel was stained with 0.1% Coomassie blue R-250, 30% methanol, and 10% acetic acid, and destained in the same solution without Coomassie blue.

### Statistics

The results are presented as means of two or three independent experiments, using cultured cells from all specimens collected from patients. Statistical analysis was performed using GraphPad PRISM5.0 (La Jolla, CA). Briefly, the Student's *t*-test was used to compare means between two independent groups whereas one-way ANOVA followed by the Tukey's post-test were used to compare means between two or more independent groups. Two-way ANOVA was used to compare group means influenced by two independent factors. The error bars represent SEM in some figures and SD in others. The level of *p* ≤ 0.05 was accepted as significant.

## CONCLUSIONS

Taken together, the most import observation in our data was the drastic difference in cell responses between the FBS and PRP supplementation, which induced us to think about improving our cell culture models to better mimic the *in vivo* environment. The proposed mechanism of PRP's action on breast tumor cells is that: 1) PRP provides the best growth factors to proliferated breast tumor cells (including TGF-β); 2) PRP mimics the network of fibrin bundles present in the breast cancer environment promoting the selection of cells with the highest potential for malignancy, activating the EMT process, and enhancing proteolytic activity; 3) PRP induces alterations in the focal adhesion complex that contributes to EMT; and 4) PRP efficiently induces endothelial cell type formation in co-cultures with breast tumor cells and HUVEC. All of these events promoted by PRP supplementation were cancer cell-type and tumor-subtype specific.

Our final consideration is that current cell culture models still need improvement to better mimic the *in vivo* environment in order to confirm if *ex vivo* results reflect the processes that occur *in vivo*. Breast cancer is a disease of multiple facets with distinct histopathological features, genetic and molecular variability, and diverse prognostic outcomes according to positive or negative ER tumor subtype. In this scenario, no single model would be capable of representing this complex disease.

In addition, no cell is isolated from other cells *in vivo*. Nevertheless, research still favors monolayers of cell lines to co-culture primary cells from patients’ biopsies. Cell lines are easier to use than primary cells. However, because they become distant from the heterogeneous tumor microenvironment while in culture, they do not display the variability of primary cells, which most likely represents the variability often observed in patients.

Primary cells derived from different patients can behave differently in culture conditions depending on the genetics and age of their corresponding patients. Moreover, fibroblasts derived from different parts of the body may have different characteristics, reflecting the beauty of biology.

This study's broadest contribution is the exploration of a cell culture condition that mimics the microenvironment of breast cancer tumors to enhance opportunities to accurately identify patients who would benefit from adjuvant therapy with target platelet functions.

### Supporting information

Supplementary Figure 1. Effects of PRP on formation of gel-like material in cell cultures. Supplementary Figure 2. Inhibition and blocking of the TGF-β Pathway in luminal B and HER2+ breast tumor cells. Supplementary Figure 3. Graphical abstract.

## SUPPLEMENTARY MATERIALS FIGURES


