# Supplementary material for: Interface between breast cancer cells and the tumor microenvironment using platelet-rich plasma to promote tumor angiogenesis - influence of platelets and fibrin bundles on the behavior of breast tumor cells
Source: Oncotarget. 2017 Feb 9;8(10):16851–74. doi: 10.18632/oncotarget.15170 (PMC5370006; doi:10.18632/oncotarget.15170)
Supplement: Supplementary file 1 [file oncotarget-08-16851-s001.pdf]

Interface between breast cancer cells and the tumor microenvironment using platelet-rich plasma to promote tumor angiogenesis - influence of platelets and fibrin bundles on the behavior of breast tumor cells

Supplementary Materials

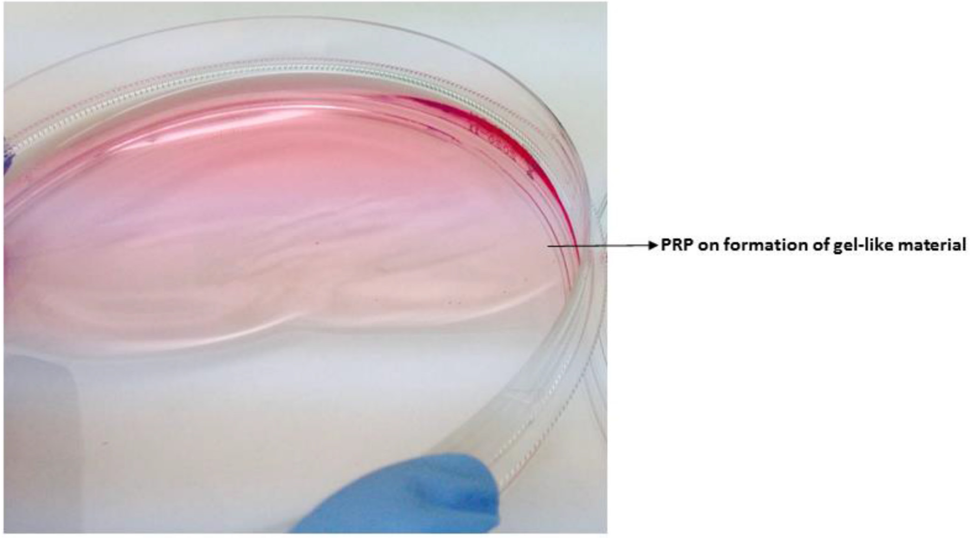

Supplementary Figure 1: Effects of PRP on formation of gel-like material in epithelial and stromal cell cultures. Cells were seeded, conditioning of epithelial and fibroblast cells began by using PRP replacing FBS. Inthiscase, 5% FBS and 5% PRP. PPP (platelet-poorplasma) was used as negative control. These conditions allowed the network of fibrinbundles to be formed in cell culture as a solid substrate and allowed the transition of cells from focal contacts in the platic surface to the fibrin bundle over the cultured cells.

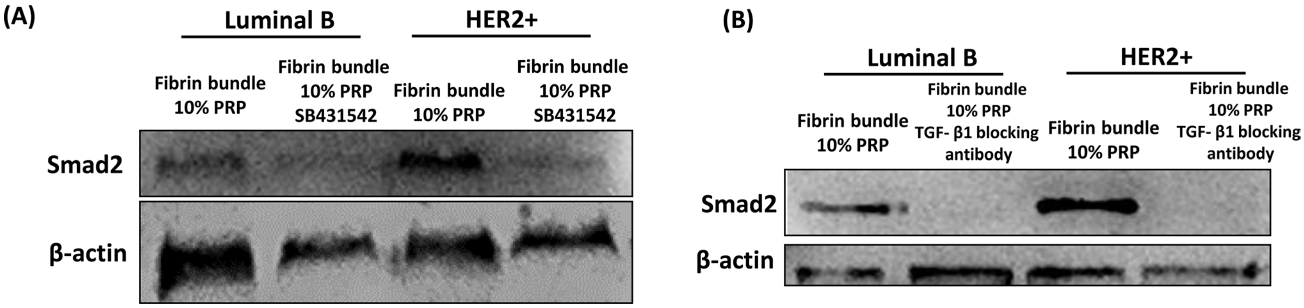

Supplementary Figure 2: Inhibition and blocking of the TGF-β Pathway in luminal B and HER2+ breast tumor cells. Detection Smad2 protein levels by immunoblotting of breast tumor cells treated with (**A**) TGFβRI inhibitor (SB431542) or a (**B**) TGFβ1 blocking antibody. β-actin is used as loading control.

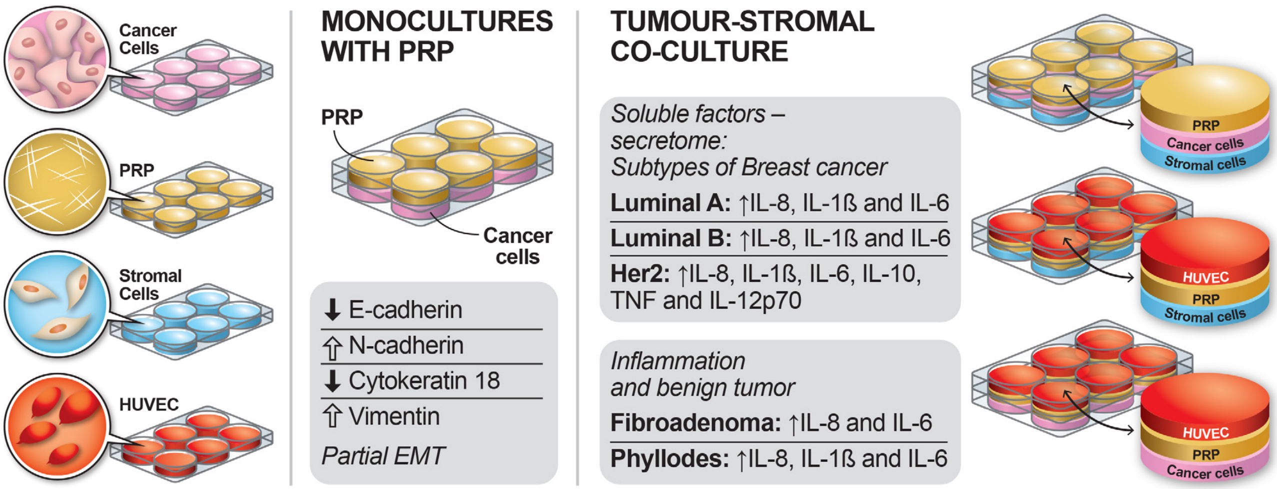

Supplementary Figure 3: Schematic drawing of the experimental procedure and results obtained with PRP.
